# The contribution of age structure to the international homicide decline

**DOI:** 10.1371/journal.pone.0222996

**Published:** 2019-10-09

**Authors:** Mateus Rennó Santos, Alexander Testa, Lauren C. Porter, James P. Lynch

**Affiliations:** 1 Department of Criminology, University of South Florida, Tampa, Florida, United States of America; 2 Department of Criminology & Criminal Justice, University of Texas at San Antonio, San Antonio, Texas, United States of America; 3 Department of Criminology and Criminal Justice, University of Maryland, College Park, Maryland, United States of America; University of Louvain, BELGIUM

## Abstract

**Background:**

Since 1990, the world’s homicide rate has declined by nearly 20%. While prior research has documented parallel homicide declines across many individual countries, the causes of a shared international homicide decline remain unknown. Drawing on a worldwide process of population ageing, and on research linking age to criminal activity, this study investigates the contribution of global demographic shifts to the international homicide decline.

**Methods:**

We draw from (1) a High Coverage Sample of 126 countries since 1990, and (2) a Long Series Sample of 26 countries since 1960 and utilize fixed-effect regressions to evaluate the impact of age structure on homicide trends. In addition, we use a quantile regression to explore variations in the relationship between age structure and homicide conditional on homicide levels.

**Findings:**

Results using the High Coverage Sample suggest no relationship between age structure and homicide. However, results from the Long Series Sample suggest that changes in the relative size of countries’ youth population is a major predictor of homicide trends since 1960. In exploring this divergence, we find that the influence of age structure on homicide becomes less evident as other risk factors for violence gain prominence. Thus, while high homicide countries had the most to gain from falling homicide rates, the safety benefits of an ageing population have been concentrated among the least violent countries.

**Interpretation:**

While the homicide declines of individual countries have often been attributed to domestic policies, the universality of international homicide trends suggests the influence of broader global phenomenon. We find that countries’ homicide trends are strongly associated with changes in the size of their youth populations, particularly where there are few competing criminogenic forces. Based on these results, we propose an explanation for the international homicide decline, while highlighting the importance of demographic patterns in explaining homicide trends.

## Introduction

Homicide is one of the world’s leading causes of premature mortality, accounting for about 400,000 deaths each year. Since the turn of the 21^st^ century, nearly every year more people have lost their lives to homicide than to war [1,2]. By 2030 homicide is expected to cause more deaths globally than infectious diseases such as tuberculosis [3]. In addition to imposing an enormous cost to human lives, homicide carries immense economic costs, estimated at approximately $171 billion in 2010 [4]. Like other public health problems, there is consensus among researchers that the causes of homicide can be identified and, at least in theory, “treated” [5]. However, social scientists still do not fully understand the causes of changes in homicide rates [6,7]. In particular, studies often attribute changes in homicide to intra-country influences, such as political, economic, and social characteristics [8,9]. The potential influence of these variables notwithstanding, there is another important shift–demographic in nature–that likely played a role in recent international trends in homicide.

**Fig 1:**
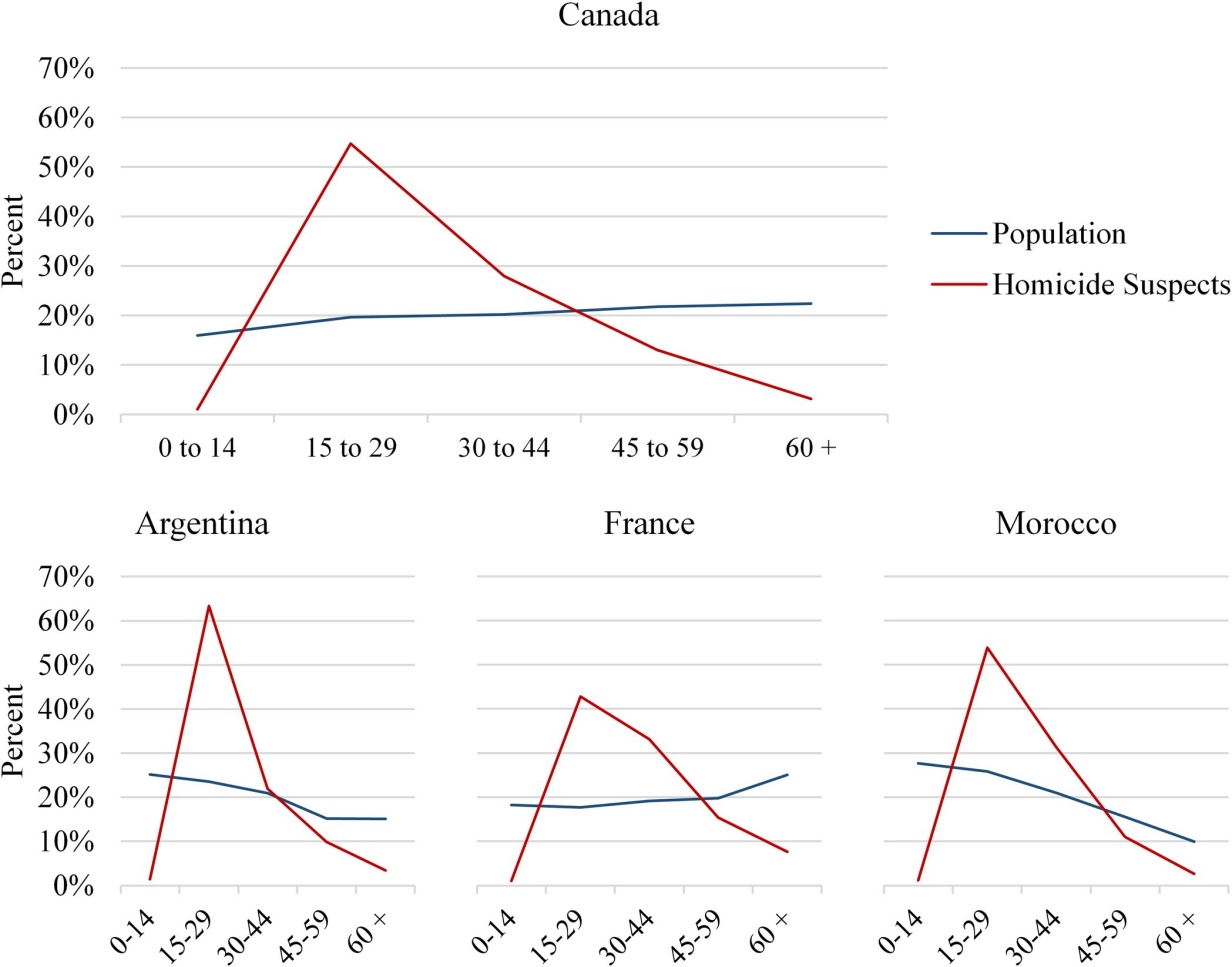
Percent distribution of persons brought into formal contact with the Criminal Justice System for homicide and of the population by age group– 2015. Shown is the age distributions of persons brought into formal contact with the Criminal Justice System for homicide (homicide suspects) and the age distribution of the population of Canada, Argentina, France and Morocco in 2015. Data are from the United Nations Office on Drugs and Crime.

In this study we investigate the contribution of age structure to international homicide trends, and the role of shifts in the age composition of populations to the international decline in homicide rates observed in recent decades. The relationship between age and criminal offending is considered a veritable fact in criminology [10,11], with violent behavior (including homicide) increasing during teenage years and then declining as individuals age into adulthood. In Canada, for example, persons between 15 and 29 years old (hereafter also referred to as youth population) make up 19.6% of the population, but represent 54.8% of all persons brought into formal contact with the criminal justice system for homicide, a concentration that is typical of other countries around the world (Fig 1). Similarly, in the United States youth between 15 and 29 years correspond to 20.9% of the total population, but are responsible for 49.8% of all arrests for violent crimes (S1 Fig). While there is growing evidence for differences in the specific age distribution of crime across countries [12,13], the overall shape of this distribution is consistent and studies regularly find that violent behavior declines as individuals age into later adulthood. Since violent crime tends to be most common amongst youth, countries with younger populations–all else equal–should have higher homicide rates [14]. Thus, we investigate the relationship between two concurrent trends: a recent decline in homicide rates across most developed countries and the aging of populations in those same countries. Further, by examining whether the role of age structure varies depending on the presence of other criminogenic forces–conditions that produce higher crime rates, such as those related to political, social, and economic distress [15]–we investigate why several of the most violent countries in the world are not experiencing declines in their rates of homicide. In short, our study extends existing knowledge by exploring the role of demographic forces for understanding international crime trends, while proposing a possible explanation for the international homicide decline.

### Age-structure and crime

Age is among the most robust predictors of criminal activity, including homicide [10,11]. Two main theoretical mechanisms predict that a larger proportion of individuals at younger ages should influence aggregate rates of homicide. First, *a simple compositional effect*—also referred to as a *simple aggregate effect*—is an extrapolation of the individual-level relationship between age and crime to the macro-level [10,15]. Since the relationship between age and criminal offending is largely consistent across individuals, an increased proportion of individuals at younger ages should increase the prevalence of potential offenders. In addition, as the lifestyle of youths place them at greater risk of violent victimization, an increase in the young population should also increase the pool of potential victims for violence [16,17]. Hence, all else equal, countries should have higher levels of violence when a greater proportion of their population is young [14].

Second, a *relative cohort size* perspective posits that that the social consequences related to age-structure stem from a disproportionally large cohort of individuals at younger ages relative to the rest of the population [18,19]. Originally, this perspective emphasized the impact of cohort size on labor market conditions, noting that youth who are members of relatively large cohorts face a range of labor market disadvantages because they generate an oversupply of labor relative to job openings. To absorb a larger cohort of workers, labor markets need to replace the jobs of individuals retiring from the market, as well as create several new positions to accommodate the incoming cohort. Hence, competition is much greater, increasing the likelihood of unemployment, and stagnating opportunities for wage growth [18].

Members of relatively large cohorts may be pushed into criminal activity by the difficulties they confront in the labor market, which may incentivize illicit methods of obtaining income while decreasing the social control provided by a legitimate job. Furthermore, these individuals may be more likely to offend because of a decrease in the number of older adults providing them with supervision and support [20,21]. For instance, a smaller adult to youth ratio may reduce the amount of monitoring available by parents and the community, as less adults will be available to give direct attention to a much larger number of youth [22]. Consequently, youth will likely spend more time with other individuals of their own age group, often without the supervision of an older adult, increasing the influence by peers within unstructured socializing settings that may be criminogenic [23]. In addition, schools, churches and other institutions that provide services to youth will be overburdened by a sudden increase in clientele, and will be more likely to have their resources overstretched. As a result, youth in larger cohorts may feel alienated and harbor feelings of pessimism and skepticism towards other segments of society [24]. Such feelings can have implications for political participation and for civil obedience, and may be aggravated by the difficulties in the labor market, and by the diminished number of interactions with individuals of other age groups.

In short, a higher proportion of youth may impact homicide rates because youth are individually more likely to be involved in violent offending (i.e. compositional effect), or because young members of larger cohorts face a range of disadvantages due to the size of their age group (i.e. relative cohort size). Many related propositions for the relationship between age composition and homicide rates can be derived from these two larger perspectives. For instance, a more youthful population may have less capital available to invest in their communities, or may be themselves less invested in their current places of residency, with consequences to the social organization of the communities where they reside [25,26]. Moreover, when organized crime and the drug trade is operated by a younger population, without the management of older adults, those illegal operations are likely to become much more lethal, as was the case with a dramatic rise in youth violence stemming from illicit-drug industry during the US crack epidemic [27]. Both the above circumstances are more likely when the population of youth is greater, and when older adults are less available.

### Worldwide trends in age structure

**Fig 2:**
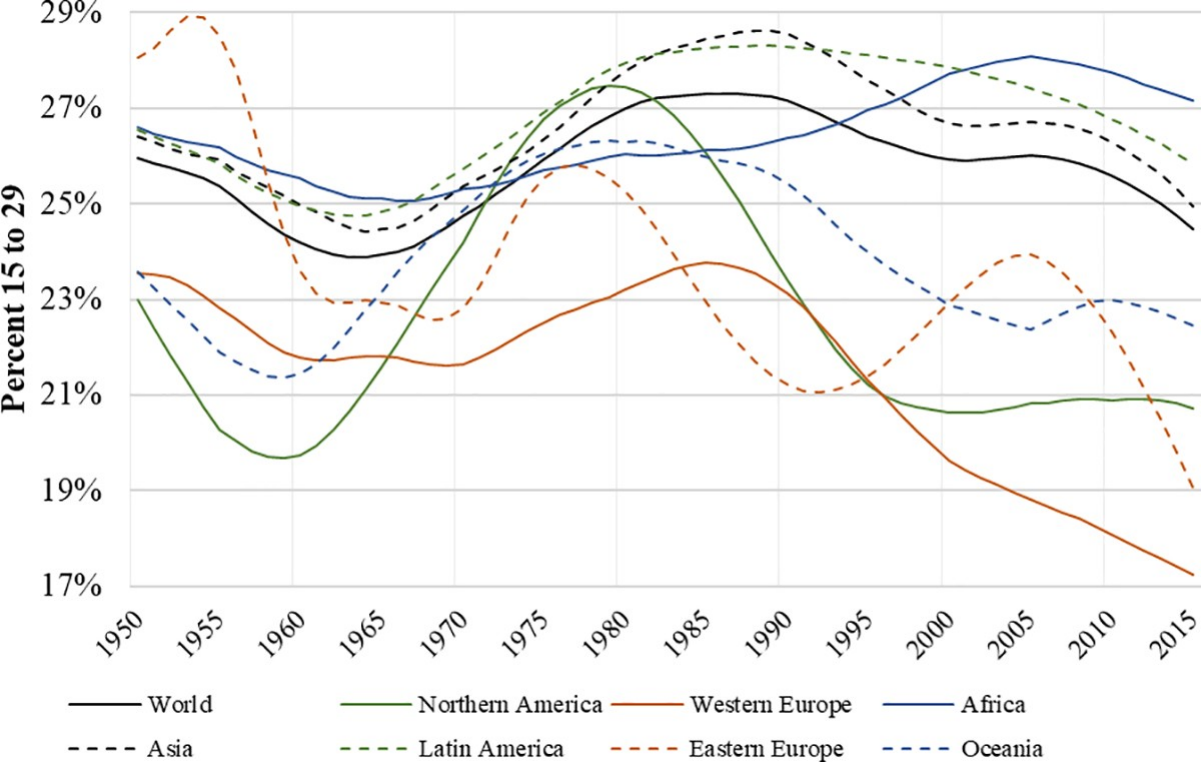
Percent of the population between 15 and 29 years by region and year– 1950 to 2015. Shown is the percentage of the population aged 15 to 29 for the entire world and across global regions from 1950 to 2015. Data are from the United Nations World Population Prospects: The 2017 Revision.

During the 1960s and 1970s most regions of the world experienced an increase in the relative size of their population 15 to 29 years old (Fig 2). The size of the youth population peaked during the late 1970s and early 1980s, when it began to drop considerably, particularly in the developed world. Then, from 1980 to 2015, all regions besides Africa experienced a decline in their youth population.

This global trend in age structure has two primary drivers: increases in life expectancy and changes in fertility patterns. Following the end of World War II, much of the world experienced a sharp, though temporary rise in fertility rates (often referred to as the “baby boom”), generating disproportionately large cohorts of newborns which profoundly affected the age-structure of their populations [28]. Simultaneously, improvements in hygiene practices and health care led to substantial drops in mortality rates across all age groups. Consequently, between 1960 and 2007 the average global life expectancy increased by more than 20 years, from 43 to 66 years [29].

During the 1960s several developing countries were also experiencing a broader demographic phenomenon called the Demographic Transition [30,31], which corresponds to a shift from high birth and high death rates to low birth and low death rates. Initially, a decrease in death rates coexist with high levels of fertility. Overtime, however, fertility rates also decline as families adjust from having many to a fewer number of children. The time difference between the decline in mortality and the decline in fertility results in a sudden increase in population size, and generates disproportionately large birth cohorts. While most developed countries began their demographic transitions in the 1800s, several developing countries experienced transitions only after World War II.

Several developed countries are also experiencing what some demographers call a Second Demographic Transition [32]. Partially driven by changes in family formation and in reproductive preferences, individuals are often delaying, or entirely avoiding parenthood [33]. Consequently, several countries are experiencing sharp declines in fertility, and many now have birth rates below the level necessary to achieve population stability [34]. Over time, this process results in a gradual decline in the overall population, while increasing the proportion of older individuals relative to the youth. Together, the aforementioned demographic processes were the catalyst of dramatic changes in the age structure of populations worldwide. Given the strong link between age and crime, these processes should be particularly consequential for homicide trends.

### Age structure and the 1990s United States’ homicide decline

Starting with the United States as an example, we note that from 1991 to 2000 the homicide rate dropped sharply by 43% from 9.7 to 5.5 homicides per 100,000 population. Such a drastic change in the homicide trend of a country the size of the United States is atypical, and suggests drastic causes. To be sure, there are no shortage of hypotheses, including the impact of gun laws, mass incarceration, the receding crack market, innovations in policing, improvements in the economy, rising incarceration rates, and the legalization of abortion [35–37]. Most of these explanations point to events or policies that are specific to the United States, but similar drops in homicide rates occurred across several countries. For example, Canada had a crime decline of similar magnitude without sharing many of the criminal justice policies particular to the United States [38,39]. In fact, several countries, especially Western democracies, experienced homicide declines of comparable or greater magnitude during the same time period [2,40–43]. If crime rates were influenced predominantly by domestic policies and local socioeconomic conditions, there should be greater divergence rather than similarity in crime trends across countries. Rather, a shared trend suggests that the homicide decline was not predominantly caused by domestic events, but by phenomena that were shared across countries.

**Fig 3:**
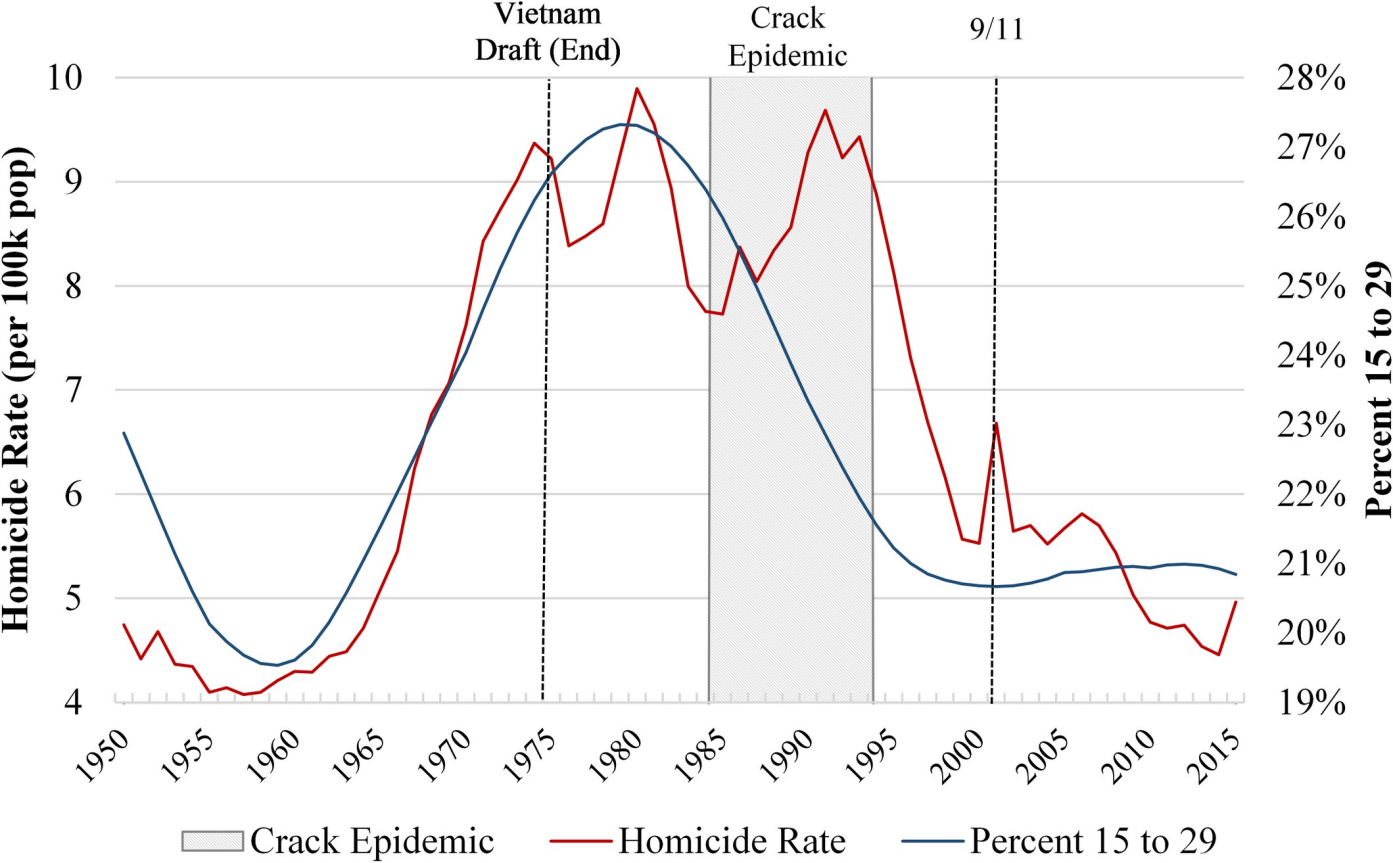
Homicide rate and percent of population 15 to 29 –United States 1950 to 2015. Shown is the annual homicide rate per 100,000 population and the percent of the population aged 15 to 29 in the United States from 1950 to 2015. Homicide data are from the United Nations Office of on Drugs and Crime Homicide Database and the World Health Organization Mortality Database. Data on the Percent 15 to 29 are from the United Nations World Population Prospects.

Fig 3 contrasts the trend in U.S. homicide rate with the percent of the population aged 15 to 29 from 1950 to 2015. During the 1960s and 1970s there is a clear positive association between youth population and increasing homicide rates, a relationship documented by prior research [14,44]. An early decline in homicide rates beginning in 1980 occurs alongside a decreasing youth population. However, this early decline was interrupted by a crime spike from 1985 to 1991. This increase has been linked to the crack epidemic, which led to an escalation of violence among youth in inner cities [27]. Nationally, the crack epidemic reverted the declining trend in homicide since 1980. However, once the epidemic receded in the early 1990s, homicide rates resumed their decline in unison with a decline in the percent of the population aged 15 to 29.

There is a remarkable similarity in the long-term trends of the homicide rate and of percent youth in the United States, a pattern which is not observed for other factors commonly associated with the 1990s homicide decline, such as improvements in the country’s economy (S2 Fig), and mass incarceration (S3 Fig). Furthermore, similar to the United States, several countries experienced a relative decline in the population between 15 and 29 years of age, concurrent with a decline in homicide (S4 Fig). Despite the strong association between age and violent criminal behavior, several recent studies have proposed that age structure is unrelated to between-country differences in homicide rates [45–47] and to the homicide declines of individual countries [36,48,49]. In contrast, we argue that age structure is associated with international homicide trends since the 1960s, including with the international homicide decline in recent decades. In doing so, we use long-term longitudinal data to examine within-country change in age-structure and homicide to assess whether changes in the size of the young population corresponds with fluctuations in a country’s homicide rate.

Moreover, prior research also demonstrates that several of most violent regions of the world are not participating in the international homicide decline [40,42,43], even though many of these countries are experiencing a decline in the size of their youth population. Accordingly, we investigate if the effect of age composition on homicide trends is less evident at times and places where other criminogenic forces are more prominent (e.g. the crack epidemic). In particular, we propose that these forces can temporarily overpower the pacifying influence of an aging population. While this proposition is a novel explanation of international homicide trends, it finds parallel in extant research at the sub-national level that has found that age composition is only related to the homicide trends in US counties where and when other criminogenic forces are absent [15]. Furthermore, several studies have documented strongest declines in violence in the safest areas of a given country, state or city, alongside a widening in the gap in the rates of violence between the most and the least violent locations [50–52]. Finally, this proposition mirrors research at the individual level, which has found difficulty in observing the influence of individual risk factors for delinquency within a sample of individuals who simultaneously concentrate too many disadvantages associated with risky behavior [53–56]. Specifically, the fact that these disadvantages tend to coexist within individuals is a challenge to researchers attempting to identify the influence of an individual risk factor, particularly when using a sample of high-risk individuals saturated with disadvantages.

Inspired by the abovementioned literature, we propose that as individuals, countries too can concentrate criminogenic disadvantages, at times at a sufficient scale to overpower the effect of demographic influences on their homicide trends. Hence, changing age-structure should have the most evident impact on homicide rates in countries with lower levels of violence, where fewer criminogenic forces are interfering with the homicide trend.

## Methods

### Data sources

Data for this study were collected from a variety of renowned international organizations including the United Nations, the World Health Organization, and the World Bank. Country-level homicides data were obtained from two sources: (1) A new edition of United Nations Homicide Statistics produced by the United Nations Office on Drugs and Crime (UNODC), which was built with the collaboration of the lead author of this study, and (2) the Mortality Database of the World Health Organization (WHO).

The United Nations Homicide Statistics is the result of a systematic consolidation of data obtained from hundreds of national and international sources, including the United Nations own archives, national police offices, health agencies, statistical agencies, as well as other international organizations (e.g. World Health Organization, Organization of American States, Eurostat). The principal source is the United Nations’ primary data collection through the United Nations Surveys on Crime Trends and the Operations of Criminal Justice Systems (UN-CTS). The UN-CTS is an annual survey of officials from UN member countries requesting answers to a series of questions related to crime and criminal justice statistics in previous years [57]. The UN-CTS also collects information regarding the data collection process and the quality of measures collected, and all data are curated through a validation process. The set of considerations related to data validation are provided in the S1 Table. According to the United Nations a homicide is defined as an “unlawful death inflicted upon a person with the intent to cause death or serious injury.” This definition includes all unlawful deaths resulting from interpersonal violence. Excluded from this definition are involuntary homicides (e.g. manslaughter), war killings, and legally justified killings such as interventions by law enforcement and homicides committed in self-defense. All data collect by the UNODC is evaluated according to its compliance with this specific definition of homicides, which is outlined in the International Classification of Crime for Statistical Purposes (ICCS). The ICCS corresponds to standards for classifying crimes which follows a strict set of statistical rules for accounting, which are independent of the legal and statistical practices of individual countries, thus ensuring the international comparability of the data. Furthermore, the UNODC works closely with countries to ensure compliance with the ICCS [58]. The UNDOC data have been used in several recent studies on cross-national homicide [59–61].

The WHO Mortality Database is an annual compilation of statistics on medically certified deaths submitted by governments to the World Health Organization. The database is a long-standing data collection effort, and includes records on over two billion deaths since 1950. Causes of deaths are classified according to the International Classification of Diseases (ICD), which defines homicide as “a death caused by injuries inflicted by another person with the intent to injure or kill, by any means, excluding injuries due to legal interventions or operations of war” [62]. The S2 Table details the classification codes that correspond to homicide across versions of the ICD.

While the UNODC and the WHO data are obtained from different sources, the definition of homicides in the ICD and the UN-CTS are largely consistent, measuring homicides as death caused by intentional acts of interpersonal violence that lack any legal justification. Because of that consistency, both the UN and the WHO make use of the other organization’s data to validate and complement their own statistics [4,63].

### Analytical samples

The current study makes use of two analytic samples: a (1) High Coverage Sample (from 1990 to 2015), and a (2) Long Series Sample (from 1960 to 2015). The High Coverage Sample includes only data from the United Nations Homicide Statistics, and covers 26 years of data for 126 of the world’s countries with more than 1 million residents in 2015. Together, these countries accounted for 90.3% of the global population in 2015, including data for virtually all regions, except parts of Africa. A world map illustrating the latest available homicide rate per 100,000 residents of each country with available data is provided in the S5 Fig.

**Fig 4:**
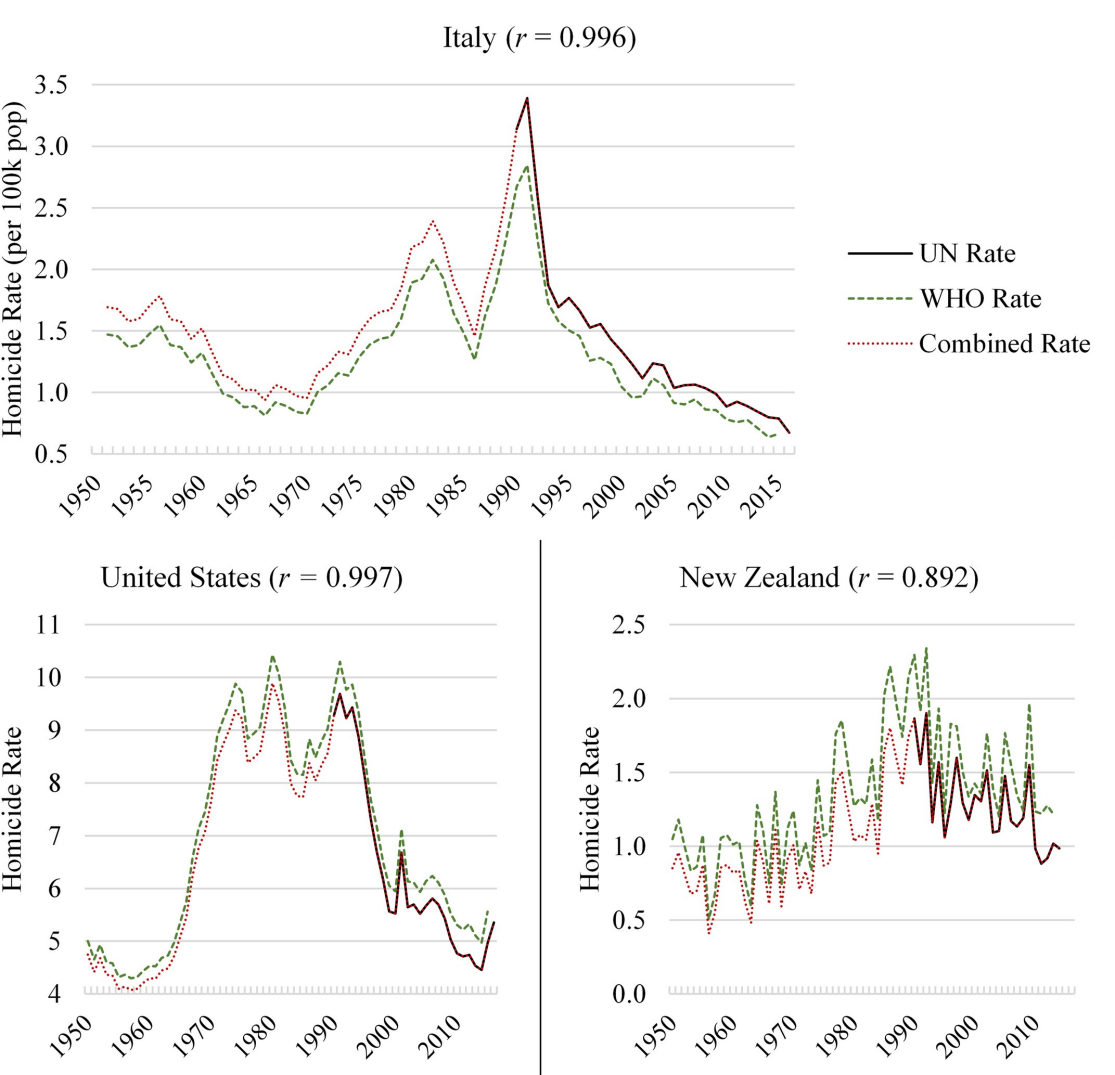
Combined homicide rate series– 1950 to 2015. Shown is the homicide rate from the United Nations Homicide Database since 1990, contrasted with the homicide rate from the WHO Mortality Database since 1950, and the Combined Series resulting from the combination between the two previous sources between 1950 and 2015 for Italy, the United States, and New Zealand. The Pearson correlation of overlapping years between the UN and the WHO homicide trends is in parenthesis.

While data from the United Nations Homicide Statistics include a broad sample of countries, these data only capture homicides since 1990. In contrast, the WHO Mortality Database captures homicide data since 1950, although for a much smaller set of countries. The Long Series Sample is the product of a combination between the UN homicide data from 1990 to 2015, with WHO homicide data from 1950 to 1989. This combined series was produced in order to extend the evaluation of the relationship between age composition and homicide trends to all the decades following World War II, when several countries experienced strong variations in their demographic compositions due to the demographic trends outlined in the section “Worldwide trends in age structure.” This combination was only executed for countries which had overlapping years of homicide data in each source, and in cases where these homicide trends displayed a strong association (i.e. Pearson correlation above 0.6). Furthermore, to account for small differences in the level of homicides between each source, WHO data were adjusted by multiplying the homicide rate in any given year by the average ratio between the WHO and the UN series. Fig 4 presents the resulting combined series using Italy, the United States and New Zealand as examples. Both the WHO and the UN homicide share similar overall trends, which is reflected in very high Pearson correlations between the series from 1990 to 2015. The dotted red lines correspond to the final combined series, which is a copy of the UN rates from 1990 to 2015 (the black lines), combined with the WHO rate from 1950 to 1989 (the dashed green line) adjusted by about 15%, -5%, and -19% respectively. This adjustment ensured a smooth continuation in the combined series, which runs for 66 years between 1950 and 2015.

The S6 Fig details the combined homicide series for the countries in the Long Series Sample. Among the 26 countries included in the Long Series Sample, the Pearson correlation between the UN and WHO rates in overlapping years between 1990 and 2015 is 0.986, which reflects the high comparability between the UNODC and the WHO data. This finding is consistent with prior research that finds a high degree of similarity between the UNODC and WHO data [64,65]. Consequently, additional analyses using only WHO homicide data in lieu of the combined series yield very similar results, which are discussed in the sensitivity analyses section.

The S3 Table reports the countries included in each sample as well as the number of years with data available, the first and last years of data availability, and the summary statistics on the homicide rate (mean, standard deviation, interquartile range, minimum and maximum). The S4 Table lists all countries and territories not included in either analytical sample. The primary reason for exclusion was missing data on homicide rates.

### Dependent variable: Homicide rate

The dependent variable is the homicide rate per 100,000 residents. Consistent with prior research, we use the natural logarithm of the homicide rate of each country and year in order to reduce the influence of extreme values of homicide rates on the estimates and to improve model fit [66]. To facilitate the interpretation of the results, we also present the exponential transformation of all coefficients, which correspond to the average proportional change in the mean homicide rate from each one-unit increase in the corresponding independent variable. The transformed coefficient can be interpreted as a percent change by subtracting its value by 1 and multiplying it by 100.

### Independent variable: Percent aged 15 to 29

Data for the percent of the population aged 15 to 29 are obtained from the United Nations World Population Prospects. Data on country-level population age structure are primarily sourced from UN member countries’ official population census records. The percent of the total population of each country-year between 15 and 29 years old was calculated by dividing the population between aged 15 and 29 years, over the sum of the population recorded in all age ranges, multiplied by 100. This measure is referred to as the percent 15 to 29, or the percent youth.

### Control variables

The analyses include controls variables for the impact of country-level social and economic characteristics that have been widely used in extant comparative homicide research [8,9]. *Gross Domestic Product* (GDP) measures the aggregated value of all goods and services produced, divided by the population of a country in a given year. To account for differences in the pricing of the same goods (e.g. inflation, exchange rates), values were adjusted to reflect the corresponding purchasing power in 2010 U.S. dollars ($). To facilitate the interpretation of coefficients, values were transformed from a scale of $1 to a scale of $1,000. The *urban population* measures the proportion of the population of a given country that resides in an urban area, as opposed to a rural area, relative to the total resident population. Data from both above indicators are collected from the World Bank Open Data. *Income Inequality* is operationalized using the GINI index obtained from the Standardized World Income Inequality Database (SWIID) [67]. The Gini index is a statistical measure of the income distribution, ranging from a hypothetical zero (i.e. perfect equality in the income of a population), to maximum value of 100 (i.e. perfect income inequality). *Percent male* measures the percentage of a country’s population that is male and is obtained from the United Nations World Population Prospects.

All data required to replicate this study are available in the S1 Dataset, which contains data in long format at the country and year level. Code for replicating or expanding the analysis is also available as a supporting document as an R Markdown file (S1 File), and as a text file (S2 File). Any additional data, code, and materials used in the analysis are available upon request.

### Descriptive statistics

The S5 Table provides the descriptive statistics of all variables by analytical sample. On average, the 26 countries in the Long Series Sample have a lower homicide rate (6.26 per 100,000) compared to the 126 countries in High Coverage Sample (8.12 per 100,000). The Long Series Sample also has lower levels of inequality, is more urbanized, and has approximately twice the GDP per capita than the High Coverage Sample. Hence, each sample has very distinct compositions, which may influence the coefficient sizes of predictors of homicides. These differences were directly addressed in our analyses below. The S6 Table provides a correlation matrix of the variables in each analytic sample. Additionally, the table contains the variance inflation factors (VIFs) of each variable in the fully controlled regression models. The VIFs in both samples remain below 3.5 suggesting no substantial problem with multicollinearity [68].

### Statistical analysis

#### Regional trends

Regional trends were calculated by aggregating the homicide and population counts of all countries within a given geographic region. Solely for the purpose of generating the regional trends, years with missing data for each country were imputed using a weighted moving average based on other years of observable data in that country’s series. In addition, the weight of each observed year on the average is weighted exponentially, meaning that the weight of each year on the average dropped by half with each year-difference from the imputation. The S7 Fig is an example of this methodology applied for Honduras. The resulting dataset with data on trends for all regions is provided as a supplementary file (S2 Dataset). Trends for Africa were not presented because of the lack of data for most of the region. Population coverage for all other regions is above 98%. Imputed data was not used for any of the regression analyses.

#### Fixed-effects regression

The average effects of changes in the percent youth on homicide trends was estimated using fixed-effects linear regression. The fixed-effects model estimates the predictors of within-country change in homicide rates overtime while controlling for any unobserved time invariant factors that may confound the relationship between age composition and homicide rates. The fixed effects model is specified using the following formula:

$${Ln(Homicide\;Rate}_{it})=\beta_{0}+\beta_{1}{Percent\;15\;to\;29}_{it}+\beta_{j}X_{it}+\alpha_{i}+\varepsilon_{it}$$

Where the left-hand side of the equation corresponds to the natural log of the homicide rate of each country *i* at time *t*. *β*_1_ represents the coefficient for the focal independent variable–percent youth–and *X_it_* represents all time-varying control variables included in the model. Next, *ε_it_* is the error term of each individual data point and *α_i_* refers to the country fixed effects, which account for country-level characteristics that are constant across time *t*. Moreover, a two-way fixed effects model—which includes a vector of controls for all years in each sample, in addition to the vector of controls for all countries—was used as an additional strategy for assessing the effect of age composition while controlling for other unobservable drivers of international homicide trends. Standard errors were clustered using the White method to account for the dependence of year-observations within each country [69]. All regression models were produced using the *plm* package in the R programming language. Models can be replicated and expanded upon using the S1 File provided as a supplementary file.

#### Quantile regression

A key proposition of this study is that the direct effect of percent youth on homicide rates is weakened in times and places where other criminogenic forces gain prominence (e.g. the US crack epidemic). This proposition implies that the observable effect of percent youth may be conditional on the absence of other major criminogenic forces. However, a challenge of addressing this empirically is that high quality data on such forces (e.g. drug epidemics, organized crime, political unrest) are typically unavailable on a global scale. To overcome this issue, we use a quantile regression (with fixed-effects) to explore differences in the effect of age composition conditional on the homicide rate of a given country. Quantile regression is a nonparametric method that enables an estimation of whether the effect of a given independent variable varies across the distribution of the dependent variable. The effect of an independent variable can be calculated conditional on any percentile (τ) of the distribution of the dependent variable thus enabling a comparison of the strength of predictors across this distribution [70,71].

In the current study, quantile regression is used to test the proposition that the observable effect of the percent youth on homicide rates is greatest in countries with lower levels of homicide, but that the influence of percent youth on homicide diminishes in countries with higher homicide rates, as other drivers of homicides gain prominence. The main advantage of using this method is that it overcomes limitations with the availability of data on other criminogenic pressures–and our lack of a comprehensive knowledge thereof—by directly using homicide rates themselves, the presumed outcome of these criminogenic forces, as an analytical strategy for evaluating variations on the impact of age composition. Quantile regression models were estimated using the *quantreg* package in R.

## Results

### Worldwide trends in homicide

**Fig 5:**
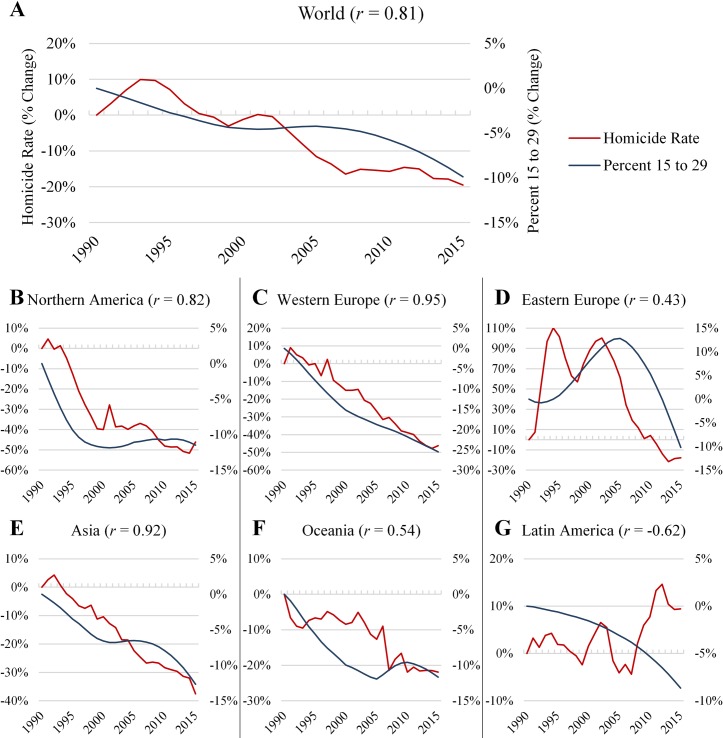
Percent change from 1990 in the homicide rate and percent of Population 15 to 29 by region– 1990 to 2015. Shown is the percent change in homicide rate and in the percent of the population 15 and 29 for each year relative to 1990, until 2015. The percent change in the percent 15 to 29 is in the secondary axis. The Pearson correlation between the two series is in parenthesis. Each region is in a part of the figure. Africa is omitted due to lack of data representing the entire region. (A) Global Trend. (B) Northern America. (C) Western Europe. (D) Eastern Europe. (E) Asia. (F) Oceania.

Since 1990, the global homicide rate is estimated to have declined by 19.7% from 6.1 per 100,000 to 4.9 per 100,000 in 2015 (Fig 5A, S8 Fig). While the homicide decline is an international event, it is not entirely global as several countries with the highest homicide rates did not experience a reduction in homicide [43]. In addition, from 1990 to 2015 the steepest drops occurred in regions already marked by lowest levels of homicide, with declines of over 40% occurring in Western Europe and Northern America, 37.5% in Asia, and both Oceania and Eastern Europe experiencing declines in homicide rates of nearly 20% (Fig 5B–5F). Across these regions, there is a notable bivariate association between trends in homicide and the percent of the population between 15 and 29 years old.

While much of the world experienced declining homicide rates beginning in the 1990s, two exceptions are of note. First, Eastern Europe experienced a large increase in homicide rates in the early 1990s, which has been attributed to the social disruption caused by the dissolution of the Soviet Union [72,73]. However, once the region stabilized in the early 2000s, homicide rates resumed a decline in unison with a reduction in the youth population (Fig 5D). Second, Latin America, which in 1990 had the world’s highest level of homicide, experienced an increase of almost 10% in its homicide rate between 1990 and 2015, despite also experiencing a decline in the proportion of its population aged 15 to 29 years (Fig 5G).

Several of the largest countries of Latin America, such as Brazil, Mexico and Venezuela, have experienced considerable increases in their homicide rates over recent decades, despite an ageing of their populations (S9 Fig). While this study does not directly investigate the reasons for high homicide rates in Latin America, many countries in the region experience inordinate levels of inequality, organized crime, economic downturn, and other sources of social and political instability [74,75]. The example of Latin America suggests that heightened levels of criminogenic forces such as these can overpower the pacifying influence of an ageing population. Much like the United States during the crack-epidemic, and Eastern Europe after the dissolution of the Soviet Union, certain period effects may temporarily disrupt the relationship between demographic forces and homicide rates, particularly when these circumstances result in sudden social change [72,76–78]. In contrast, where such criminogenic forces are scarce, or once they recede, the relationship between demographic pressures and homicide rates becomes most notable. This variation in the effect of age composition could serve as an explanation for the international homicide decline, while also providing an answer as to why the most violent locations are not experiencing declines in their homicide rates [40,43], as other criminogenic forces impacting those locations may be preventing them from accruing the safety benefits of the ageing of their populations.

### The average impact of age composition on homicide trends

Results of the fixed-effects linear regression using the High Coverage Sample of 126 countries beginning in 1990, suggest that the association between percent youth and changes in homicide net of control variables is approximately zero (Table 1A). Next, we estimate the relationship between percent youth and homicide using the 26 countries of the Long Series Sample, which begins in 1960 (Table 1B). These results demonstrate that a one percentage point increase in the percent youth is associated with an increase in the homicide rate by 5.4%. This suggests a strong association between changes in age composition and homicide trends exist in the Long Series Sample such that, a country which experienced a decline of 6.4 percentage points in the proportion of its population between 15 and 29 years old (as did the United States between 1980 and 2010), is predicted to experience a 28.6% reduction in its (unlogged) homicide rate.

**Table 1:** Fixed effects models for the average effect of percent 15 to 29 on (ln) homicide rate.

**A. High Coverage Sample (Since 1990)**							
	coef	exp(coef)	std err	t-score	P>|z|	[95.0% Conf. Int.]	
**Percent 15 to 29**	**0.018**	**1.018**	**0.015**	**1.178**	**0.239**	**0.048**	**-0.012**
Percent Male	0.032	1.032	0.053	0.603	0.547	0.135	-0.072
Gini Index	-0.011	0.989	0.016	-0.687	0.492	0.021	-0.043
GDP per Cap (1k)	-0.031	0.969	0.010	-3.106	0.002	-0.011	-0.051
Percent Urban	0.008	1.008	0.009	0.873	0.383	0.026	-0.010
**B. Long Series Sample (Singe 1960)**							
	coef	exp(coef)	std err	t-score	P>|z|	[95.0% Conf. Int.]	
**Percent 15 to 29**	**0.053**	**1.054**	**0.014**	**3.738**	**0.000**	**0.080**	**0.025**
Percent Male	0.118	1.125	0.075	1.561	0.119	0.265	-0.030
Gini Index	-0.033	0.967	0.019	-1.794	0.073	0.003	-0.070
GDP per Cap (1k)	-0.003	0.997	0.006	-0.614	0.539	0.008	-0.014
Percent Urban	0.022	1.022	0.009	2.376	0.018	0.040	0.004
**C. Long Series Sample (Since 1990)**							
	coef	exp(coef)	std err	t-score	P>|z|	[95.0% Conf. Int.]	
**Percent 15 to 29**	**0.056**	**1.058**	**0.021**	**2.711**	**0.007**	**0.097**	**0.016**
Percent Male	0.127	1.135	0.078	1.625	0.105	0.280	-0.026
Gini Index	-0.039	0.961	0.035	-1.110	0.268	0.030	-0.109
GDP per Cap (1k)	-0.013	0.987	0.010	-1.405	0.161	0.005	-0.032
Percent Urban	0.015	1.015	0.016	0.935	0.350	0.047	-0.017

Shown are the results from fixed effects regression models estimating the natural log of homicide rates as a function of percent 15 to 29 and other control variables. (A) Model using the High Coverage Sample since 1990 (2,283 observations, 126 countries, r-squared = 0.125). (B) Model using the Long Series Sample since 1960 (1,136 observations, 26 countries, r-squared = 0.259). (C) Model using the Long Series Sample since 1990 (662 observations, 26 countries, r-squared = 0.282).

Next, we investigate the reasons for divergence in the results across the two samples. Given the differences in the composition of the High Coverage Sample and Long Series Sample in regard to both the number of countries and the number of years, it is possible that the divergence in results between two samples is driven by differences in either (1) the composition of countries in each sample (i.e. 126 versus 26 countries), or by (2) differences in time frames (i.e. since 1990 versus since 1960). To assess these possibilities, we restricted the fixed-effects regression of the Long Series Sample to data since 1990 (Table 1C). As effect sizes of this model are similar to those obtained using the original Long Series Sample since 1960, differences in results between the Long Series and High Coverage sample are likely a consequence of the differences in the composition of countries in each sample, rather than of the changes to the time frame. In particular, the High Coverage Sample concentrate substantially higher levels of social and economic disadvantages when compared to the Long Series Sample, including by having, on average, significantly more inequality in income, a 43% lower GPD per capital, and 30% more homicides relative to population size (S5 Table).

Next, Table 2 presents these same sets of analysis using a two-way fixed effect model. The coefficients for the percent 15 to 29 are remarkably similar between the one-way fixed effects models (just country), and the two-way fixed effects models (country and year). The most substantial difference is an increase in the standard error associated with the coefficient of age composition in the two-way model using the Long Series Sample since 1990 (Table 2C), which increases by 33% (from 0.021 to 0.028). That increase suggests that the addition of the year fixed effects may lead to an overspecification of that model, particularly due to a reduction in degrees of freedom. Regardless, results from the two-way fixed effects models generally add support to the finding that an effect of age composition emerges when evaluated using the Long Series Sample not because of the size of that series, but due to the composition of countries in that sample.

**Table 2:** Two-way fixed effects (country and year) models for the average effect of percent 15 to 29 on (ln) homicide rate.

**A. High Coverage Sample (Since 1990)**							
	coef	exp(coef)	std err	t-score	P>|z|	[95.0% Conf. Int.]	
**Percent 15 to 29**	**0.017**	**1.017**	**0.014**	**1.192**	**0.233**	**0.045**	**-0.011**
Percent Male	0.019	1.019	0.043	0.430	0.667	0.103	-0.066
Gini Index	-0.020	0.980	0.015	-1.335	0.182	0.009	-0.049
GDP per Cap (1k)	-0.010	0.990	0.010	-1.042	0.298	0.009	-0.030
Percent Urban	0.028	1.028	0.010	2.730	0.006	0.048	0.008
**B. Long Series Sample (Singe 1960)**							
	coef	exp(coef)	std err	t-score	P>|z|	[95.0% Conf. Int.]	
**Percent 15 to 29**	**0.051**	**1.052**	**0.019**	**2.681**	**0.007**	**0.088**	**0.014**
Percent Male	0.120	1.128	0.059	2.032	0.042	0.236	0.004
Gini Index	-0.038	0.962	0.020	-1.934	0.053	0.001	-0.077
GDP per Cap (1k)	-0.003	0.997	0.010	-0.247	0.805	0.018	-0.023
Percent Urban	0.023	1.024	0.007	3.211	0.001	0.038	0.009
**C. Long Series Sample (Since 1990)**							
	coef	exp(coef)	std err	t-score	P>|z|	[95.0% Conf. Int.]	
**Percent 15 to 29**	**0.050**	**1.051**	**0.028**	**1.744**	**0.082**	**0.105**	**-0.006**
Percent Male	0.126	1.134	0.067	1.895	0.059	0.257	-0.004
Gini Index	-0.043	0.958	0.040	-1.073	0.284	0.035	-0.121
GDP per Cap (1k)	-0.009	0.991	0.014	-0.657	0.511	0.018	-0.037
Percent Urban	0.021	1.021	0.017	1.232	0.218	0.054	-0.012

Shown are the results from fixed effects regression models estimating the natural log of homicide rates as a function of percent 15 to 29 and other control variables. (A) Model using the High Coverage Sample since 1990 (2,283 observations, 126 countries, r-squared = 0.072). (B) Model using the Long Series Sample since 1960 (1,136 observations, 26 countries, r-squared = 0.217). (C) Model using the Long Series Sample since 1990 (662 observations, 26 countries, r-squared = 0.130).

### Differences in effect by level of homicide

**Fig 6:**
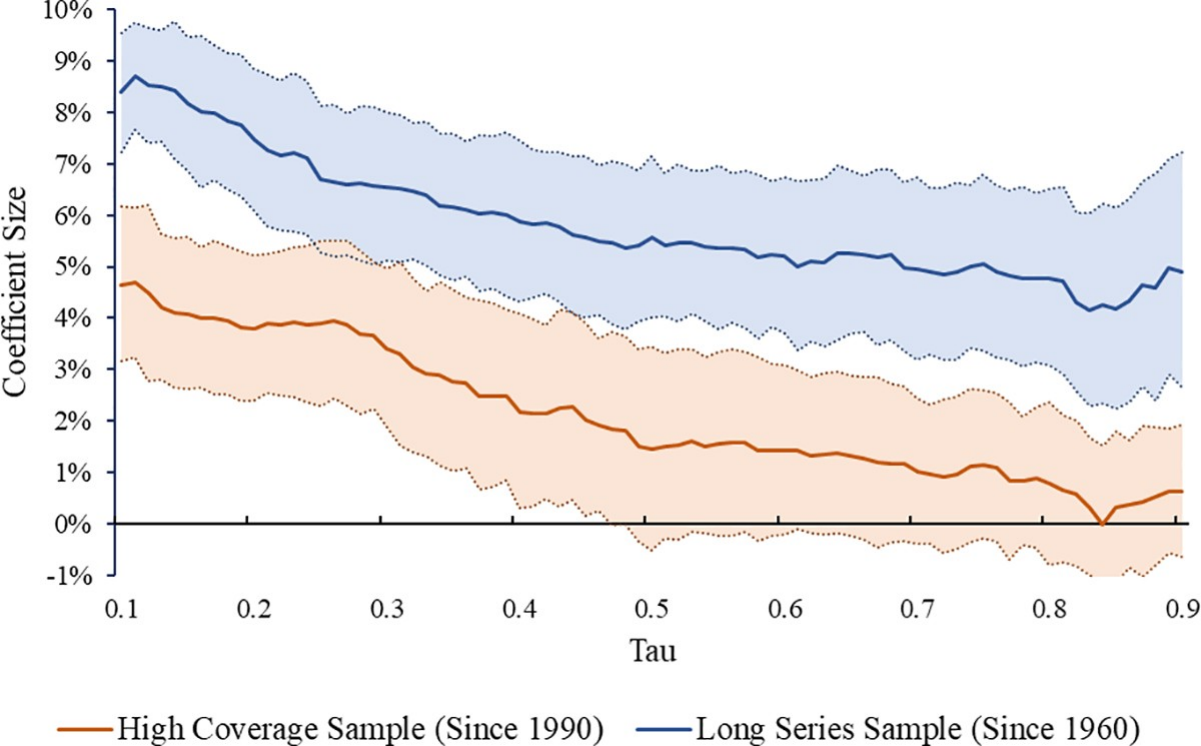
Coefficient of the percent 15 to 29 by homicide rate percentile. Shown is the coefficient of the percent 15 to 29 as a predictor of homicide rate in a quantile regression with fixed effects for each percentile (τ) of the distribution of homicide rate. Greater values of τ reflect the effect of percent 15 to 29 on homicide rates conditional on a higher level of homicides. Confidence intervals correspond to an alpha level of 0.95. One series is presented for each the High Coverage and the Long Series samples.

We estimated a series of quantile regressions to measure the relationship between percent youth and homicide rates conditional on each percentile of the homicide rate distribution (Fig 6, S7 and S8 Tables). In both the Long Series and in the High Coverage Samples, the association between age composition and homicide is strongest for countries with the lowest homicide rates. Results suggest that, for the countries at the 1^st^ decile of the homicide rate distribution (the safest of the world; tau = 0.1), a one percentage point increase in the population 15 and 29 years old corresponds to an increase of approximately 4.6% in the homicide rate. The impact of age, however, becomes gradually smaller at higher levels of the homicide distribution, and near the median of the homicide distribution of the High Coverage Sample, the confidence interval of the coefficient for percent youth crosses zero. For the Long Series Sample, the overall trend follows approximately the same pattern. However, because the Long Series sample is composed of countries with lower levels of homicide, the effect of percent youth is greater for the Long Series sample relative to the High Coverage Sample.

In short, the estimated effect of percent youth on homicide trends decline as country-level homicide rates increase. We interpret these results as supportive of the conclusion that age-structure exerts a major influence on homicide trends, and serves as an explanation for the international homicide decline which occurred across the safest countries in the world. However, the relationship between age structure and homicide becomes less evident in countries with higher levels of homicide.

### Sensitivity analyses

We conducted a series of sensitivity analyses to assess the robustness of the results. First, analyses of the current study were also performed replacing the population between 15 to 29 years of age with the population between 15 to 24 years of age. Both measures of the size of the youth population are very highly correlated (*r* = 0.924), and results of analyses using either measure are substantively the same (S9 Table). Second, to assess the influence of potential outliers, we re-estimated the models using a robust regression, which applies a smaller weight to observations with a higher residual, thus reducing the influence of these observations on the estimates [79,80]. The results of these models provide substantively similar results to our original models suggesting that the core findings of this study are not driven by outlier observations (S10 Table). Third, as a test of the robustness of our combined series we performed a sensitivity analysis contrasting results of our analyses using the combined WHO and UNODC Series since 1960, against analyses only using the original WHO homicide data since 1960 for the 26 countries of the Long Series Sample. These analyses yield virtually the same results (S11 Table). Fourth, we conducted an additional analysis to assess whether the results were influenced by missing data on the control variables. In the main analysis, listwise deletion was used to handle missing data. To assess whether missing data biased the estimates, we conducted an additional analysis estimating the bivariate relationship between percent youth and homicide using all country-year observations available with data on homicide and on the percent youth. This increases the sample size to *n* = 2,588 and *n* = 1,621 for the High Coverage sample and Long Series sample respectively by retaining cases that were dropped when control variables were included in the model. The results presented in the S12 Table demonstrate that the coefficient sizes of the percent youth using the larger samples are substantively similar to those reported in the main analysis. This analysis suggests that the core findings are not substantively affected by changes in the sample composition due to missing data. Fifth, statistical power analysis performed using the software G*Power 3.1 indicate that, regardless of the nested structure of our data, less than 100 observations would be necessary to adequately estimate the coefficient for age composition [81]. The smallest of our analytical samples (the Long Series since 1990) has 662 observation.

Finally, we conducted several analyses examining alternative operationalizations of demographic composition, particularly by using measures of relative cohort size. We found that increases in the absolute size (i.e. the raw count) of the youth population has no association with the homicide trend (S13 Table), unless a control variable is also included for the total size of the population (S14 Table). Hence, an increase in the absolute size of the youth population is only impactful for the homicide trend if the total population is held constant–an analysis analogous to the main models of this study that considers the impact of size of the youth population relative to the size of the total population. Next, we executed additional analysis replacing the percent 15 to 29 (population 15 to 29 over the total population) with alternative measures of the size of the youth cohort relative to older age groups only, namely ages 15–29 relative to ages 30–59, and ages 15–25 relative to ages 25–59. Furthermore, we also included a model utilizing the number of males ages 15–29 over the total population. While the average association between all age composition measures and the homicide rate is remarkably similar, there is a notable increase in the variability of the coefficients of the two first alternatives when compared to the standard error of the original age composition measure (S15 Table). Hence, results suggest that the percent of the population between 15 to 29 years of age is more suitable for identifying the effect of age composition on homicide trends than alternative measures of relative cohort size. Results utilizing the combined male youth measure are substantively similar to those utilizing the original percent youth measure. The coefficient for the percent males 15–29 is higher than the coefficient for the percent 15–29, a consequence of the fact that the later model does not include a measure of percent male (which is positive in the original model), and of the fact that it potentially confounds an effect of age composition with sex composition.

## Discussion

Many countries have experienced substantial declines in homicide rates over the previous decades. While several studies have documented the existence of an international homicide decline [2,40–43], no research to date has provided a comprehensive explanation for why it occurred. In the current study, we shifted the focus away from domestic policies and social events within individual countries to a broader global phenomenon, and argue that changes in country-level age structure is a key factor in understanding global homicide trends over the past six decades.

Evidence presented in the current study supports the conclusion that the ageing of populations globally has been a driver of the international homicide decline, and that the percentage of a country’s population aged 15 to 29 is strongly associated with global homicide trends since the 1960s. While global homicide rates are already at the lowest levels in decades, our results suggest that as populations continue to grow older in the future, the world may expect to see continued reductions in homicide rates. However, while an aging population may result in a desirable reduction in violence, it also creates causes for concern, including fewer individuals of working-age and more elderly in need of support [82].

Our findings also indicate that the significance of age structure on driving the homicide trend is not universal. Rather, this study demonstrated that the influence of age structure on homicide trends is less apparent in countries with high levels of homicide. Our findings at the country-level mirror several recent sub-national analyses which find that violence reductions in recent years have largely occurred in safer geographic areas to begin with, whereas areas that were exposed to the most violence before crime declines began largely remained the most violent afterward [15,50–52]. Thus, while high homicide countries have the most to gain from falling homicide rates, the safety benefits of an ageing population have been concentrated in the least violent countries, resulting in even greater disparity between the homicide rates of the safest and of the most violent regions in the world.

We propose that the differential participation of countries on the international homicide decline may in part be the result of a saturation effect, such that the influence any single driver of homicides (i.e. age structure) may become saturated in places or periods when many other risk factors for violence are most prominent. Thus, even though population aging may be placing downward pressure in the homicide rates of even the most violent regions of the world, that aging effect may be of little importance in driving the overall homicide trend in places facing the most severe societal challenges. In those countries an aging population may not be able to compete with the countervailing forces of drug epidemics, economic downturn, increased inequality, social and political instability, organized crimes, ineffective governments, and a range of other issues which support the high levels of violence of certain areas.

This finding is also consistent with research at the individual level which demonstrates that the observable effect of risk factors for offending becomes less clearly observable when individuals have a concentration of disadvantages [53–56]. Consequently, the effect of each individual risk factor is most clear when the concentration of other disadvantages is low. In this study, we suggest that countries too can be saturated of disadvantages which are highly criminogenic, and which must be accounted for when evaluating the drivers of homicide trends.

Notably, Latin American a region has experienced increasing homicide rates alongside a reduction in the size of its youth population. Much of the surge in violence within this region has been attributed to the escalating drug wars and the position of these countries as key producers and transit points for the illicit narcotics [83]. Particularly in countries with the highest crime rates, the drug trade operates in combination with organized crime groups which constitute a major source of political instability, and which largely inhibit the ability of the state to manage the violence these groups generate. In many aspects, violence in Latin America often resembles armed conflicts, both in the level or organization of its perpetrators, which are frequently members of very large groups–often with political leverage–and in the scale of the lethality these groups generate [84]. Moments of peak violence in countries across the region often occurred when political instability was also at its highest, such as the Colombian Conflict, the drug wars in Honduras, the recent political and economic crises in Brazil, and the present-day instability in Venezuela. As Nivette (p. 143) notes: “paramilitary groups, drug traffickers, and youth gangs have actively challenged the sovereignty of the state” [85].

Aside from direct violence related to drug trafficking, many high-violence countries in the region are also marked by high levels of corruption, lack of professionalism in government and by the inefficacy of their criminal justice organizations–which are often unable the handle the volume of crime within their jurisdictions [86]. Consequently, perceptions of police corruption are greater and the ability of police to control and provide security are lower in Latin America compared to Western countries [85], further incentivizing the use of violence for the resolution of private conflicts. In short, many Latin American countries are characterized by a preponderance of severe criminogenic factors including high rates of economic inequality, poverty, abundance of firearms, drug-trafficking, organized crime, government ineffectiveness, and others [74,75,84,85,87]. Accordingly, while demographic factors may be key in explaining many of the social changes in the region, population aging may be insufficient to produce a distinguishable change on homicide trends in the presence of multiple other criminogenic disadvantages.

Overall, the findings of this study hold several important implications. First, demographic patterns deserve special attention in explaining homicide trends. Importantly, while such a relationship may seem unsurprising, our findings stand in contrast to research suggesting that age structure is unrelated to country-level crime variation [36,45–48]. Moving forward, researchers should take advantage of the link between age and crime for developing theories to explain changes in crime and for developing methods to forecast future trends. Our findings also hold implications for public officials. In many countries, policymakers often link declining homicide trends to the implementation of their own domestic policies, some of which can be highly restrictive of individual’s freedoms [88]. To be clear, we do not argue that crime policies are not meaningful, rather we suggest that the evaluation of such policies must also account for the influence of broader shifts in demographic patterns. Finally, our research also encourages a focus on evidenced based policies and programs which provide support to at-risk youth as a strategy for violence prevention. Examples include the Nurse-Family Partnership [89], a program for supporting low-income mothers and newborns, and the Multisystemic Therapy [90], an intensive family and community based treatment program targeting serious juvenile offenders. These and numerous other initiatives have been demonstrated to be effective in interrupting deviant trajectories and in reducing the vulnerability of high risk youth even before the onset of serious violent behavior [89–92]. Given the strong link between the size of the youth population and homicide rates, such programs may have the potential to substantially enhance public safety, if implemented properly and at a large enough scale [93,94].

## Supporting information

S1 Fig Violent crime arrestees and population by age group–United States, 2015. Shown is the age distributions of arrestees for violent crimes in the United States, and of percent of the United States population by age group in 2015. Arrest data are from the Uniform Crime Report of the United States Federal Bureau of Investigation. Population data are from the United Nations World Population Prospects.
(PDF)

S2 Fig Homicide rate and GDP per capita–United States, 1950 to 2016. Shown is the annual homicide rate per 100,000 population and the percent of the Gross Domestic Product (GDP) in the United States from 1950 to 2015. Homicide data are from the United Nations Office of on Drugs and Crime Homicide Database and the World Health Organization Mortality Database. Data on the GDP are from the World Bank and corresponds to constant 2010 US$.
(PDF)

S3 Fig Homicide rate and prison rate–United States, 1950 to 2016. Shown is the annual homicide rate per 100,000 population and the percent of the Gross Domestic Product (GDP) in the United States from 1950 to 2015. Homicide data are from the United Nations Office of on Drugs and Crime Homicide Database and the World Health Organization Mortality Database. Data on the prison rate are from the United States Bureau of Justice Statistics.
(PDF)

S4 Fig Homicide rate and percent of population 15 to 29 –High correlations, 1950 to 2016. Shown is the annual homicide rate per 100,000 population and the percent of the population aged 15 to 29 in the Canada, Austria, and Japan from 1950 to 2015. Homicide data are from the United Nations Office of on Drugs and Crime Homicide Database and the World Health Organization Mortality Database. Data on the Percent 15 to 29 are from the United Nations World Population Prospects.
(PDF)

S5 Fig World map of homicide rates. The map is an illustration and makes no political statement. Some countries or entities are too small to be visible in the map. This map was created using the website mapchart.net. Reprinted from https://mapchart.net/world.html under a CC BY license, with permission from Minas Giannekas (owner and creator of the map-making website mapchart.net).
(PDF)

S6 Fig Homicide rate trend by source for countries with combined series. The Ratio corresponds to four-year average between the WHO homicide rate and the UN homicide rate between 1990 and 1993. The correlation corresponds to the Pearson correlation between the both rates over all overlapping years in the two series.
(PDF)

S7 Fig Moving average estimate of the homicide trend–Honduras, 1990 to 2015. Shown is the observed and estimated homicide rate for Honduras from 1990 to 2015. The redline and open circles correspond to the estimated rate using an exponentially weighted average. The blackline and closed circles correspond to the observed homicide rate. Homicide data are from the United Nations Office of on Drugs and Crime Homicide Database.
(PDF)

S8 Fig Homicide rate and percent of Population 15 to 29 by region– 1990 to 2015. Shown is the homicide rate and the percent of the population 15 and 29 from 1990 and 2015. The percent 15 to 29 is in the secondary axis. Both axis in this figure correspond to the actual values of the homicide rate and of the percent 15 to 29 in each year, and not to the change in these series from 1990. The Pearson correlation between the two series is in parenthesis. Each region is in a part of the figure. Africa is omitted due to lack of data representing the entire region. (A) Global Trend. (B) Northern America. (C) Western Europe. (D) Eastern Europe. (E) Asia. (F) Oceania.
(PDF)

S9 Fig Homicide rate and percent of population 15 to 29 –Low correlations, 1950 to 2016. Shown is the annual homicide rate per 100,000 population and the percent of the population aged 15 to 29 for Brazil, Mexico, and Venezuela from 1950 to 2015. Homicide data are from the United Nations Office of on Drugs and Crime Homicide Database and the World Health Organization Mortality Database. Data on the Percent 15 to 29 are from the United Nations World Population Prospects.
(PDF)

S1 Table Considerations for data validation in the United Nations Homicide Statistics. (PDF)

S2 Table Classification codes for homicides in the WHO Mortality Database by version of the International Classification of Diseases (ICD). (PDF)

S3 Table List of countries with summary of data availability. (PDF)

S4 Table List of countries absent from the analytical samples. (PDF)

S5 Table Descriptive statistics by sample. Shown is the total sample size (N), mean, standard deviation (SD), interquartile range, minimum, maximum values, and year range of available data for all variables included in the High Coverage Sample and Long Series Sample.
(PDF)

S6 Table Correlation matrix per sample with variance inflation factor. Shown is the personal correlation coefficient and variance inflation factor (VIF) for all variables in the High Coverage Sample and Long Series Sample. The VIFs are is based on a fixed effects model predicting the Natural Log of Homicide Rate, controlling for all other variables in the matrix, for each sample.
(PDF)

S7 Table Quantile regression with fixed effects for the effect of percent 15 to 29 on (ln) homicide rates–High Coverage sample (since 1990). Shown are the coefficients from the quantile regression model with fixed effects for the High Coverage Sample. Coefficients are exponentiated and correspond to the average proportional change in the homicide rate from a one-unit increase in the corresponding independent variable. In parenthesis are standard errors clustered by country. ***p < 0.001; **p < 0.01; *p < 0.05.
(PDF)

S8 Table Quantile regression with fixed effects for the effect of percent 15 to 29 on (ln) homicide rates–Long Series sample (since 1960). Shown are the coefficients from the quantile regression model with fixed effects for the Long Series Sample. Coefficients are exponentiated and correspond to the average proportional change in the homicide rate from a one-unit increase in the corresponding independent variable. In parenthesis are standard errors clustered by country. ***p < 0.001; **p < 0.01; *p < 0.05.
(PDF)

S9 Table Sensitive analysis—Fixed effects models for the average effect of percent 15 to 24 on homicide rate. Shown are the results from fixed effects regression models estimating the natural log of homicide rates as a function of percent 15 to 24 and other control variables. Coefficients are exponentiated and correspond to the average proportional change in the homicide rate from a one-unit increase in the corresponding independent variable. In parenthesis are robust standard errors clustered by country. ***p < 0.001; **p < 0.01; *p < 0.05.
(PDF)

S10 Table Sensitive analysis–Robust regression models with fixed effects (Huber weights) for the average effect of percent 15 to 29 on homicide rates. Shown are the results from fixed effects regression models estimating the natural log of homicide rates as a function of percent 15 to 29 and other control variables. Huber weights were used to reduce the influence of observations with high residuals on the regression estimates. Coefficients are exponentiated and correspond to the average proportional change in the standard deviation of the homicide rate from a one-unit increase in the standard deviation of the corresponding independent variable. In parenthesis are robust standard errors clustered by country. ***p < 0.001; **p < 0.01; *p < 0.05.
(PDF)

S11 Table Sensitive analysis comparing results of the Long Series sample using the combined UN & WHO series, against the WHO series alone. Shown are the results from fixed effects regression models estimating the natural log of homicide rates as a function of percent 15 to 29 and other control variable using the Combined WHO and UNODC Series since 1960, compared to only WHO data since 1960 for the 26 countries of the Long Series Sample. Coefficients are exponentiated and correspond to the average proportional change in the homicide rate from a one-unit increase in the corresponding independent variable. In parenthesis are robust standard errors clustered by country. ***p < 0.001; **p < 0.01; *p < 0.05.
(PDF)

S12 Table Sensitive analysis restricting the bivariate models to the fully controlled sample of observations. Shown are the fixed effects regression estimates using the High Cover and Long Series Sample using all observed data on Homicide Rate and Percent 15 to 29. Coefficients are exponentiated and correspond to the average proportional change in the homicide rate from a one-unit increase in the corresponding independent variable. In parenthesis are standard errors clustered by country. ***p < 0.001; **p < 0.01; *p < 0.05.
(PDF)

S13 Table Sensitive analysis—Fixed effects models for the average effect of the absolute size of the population 15 to 29 on homicide rate. Shown are the results from fixed effects regression models estimating the natural log of homicide rates as a function of the absolute size of the population 15 to 29 (in millions of individuals) and other control variables. Coefficients are exponentiated and correspond to the average proportional change in the homicide rate from a one-unit increase in the corresponding independent variable. In parenthesis are robust standard errors clustered by country. ***p < 0.001; **p < 0.01; *p < 0.05.
(PDF)

S14 Table Sensitive analysis—Fixed effects models for the average effect of the absolute size of the population 15 to 29 on homicide rate controlling for total population. Shown are the results from fixed effects regression models estimating the natural log of homicide rates as a function of the absolute size of the population 15 to 29 (in millions of individuals) and other control variables, included the total size of the population (in millions of individuals). Coefficients are exponentiated and correspond to the average proportional change in the homicide rate from a one-unit increase in the corresponding independent variable. In parenthesis are robust standard errors clustered by country. ***p < 0.001; **p < 0.01; *p < 0.05.
(PDF)

S15 Table Sensitive analysis—Fixed effects models for the average effect of youth cohort size relative to older age groups. Shown are the results from fixed effects regression models estimating the natural log of homicide rates as a function of the size of the youth cohort relative to older age groups, and as a function of males 15 to 29 years over the total population. The model including the original measure of percent youth (ages 15 to 29 over the total population) is in the and fifth columns. To account for differences in the effective range of each age measure and to enable a direct comparison between coefficients, all measures included in the model were standardized. Coefficients are exponentiated and correspond to the average proportional change in the standard deviation of the homicide rate from a one-unit increase in the standard deviation of the corresponding independent variable. In parenthesis are robust standard errors clustered by country. ***p < 0.001; **p < 0.01; *p < 0.05.
(PDF)

S1 Dataset Country and year level dataset. This file contains the data used to produce all analyses of the current study, organized in long format by country and year. The first sheet contains all data, and the second sheet contains a description of each column.
(XLSX)

S2 Dataset Region and year level dataset. This file contains data at the regional and global level, resulting from the aggregation of country-level data. Data is organized in long format by region and year. The first sheet contains all data, and the second sheet contains a description of each column.
(XLSX)

S1 File Code for processing the data and replicating the analyses. The code was originally written as a R markdown file. Any other materials using in this study are available upon request.
(RMD)

S2 File Code for processing the data and replicating the analyses in text format. This file is a copy of S1 File in .txt format.
(TXT)
