# Supplementary material for: The contribution of age structure to the international homicide decline
Source: PLoS One. 2019 Oct 9;14(10):e0222996. doi: 10.1371/journal.pone.0222996 (PMC6784918; doi:10.1371/journal.pone.0222996)

S1 Fig. Violent crime arrestees and population by age group – United States, 2015. Shown is the age distributions of arrestees for violent crimes in the United States, and of percent of the United States population by age group in 2015. Arrest data are from the Uniform Crime Report of the United States Federal Bureau of Investigation. Population data are from the United Nations World Population Prospects.

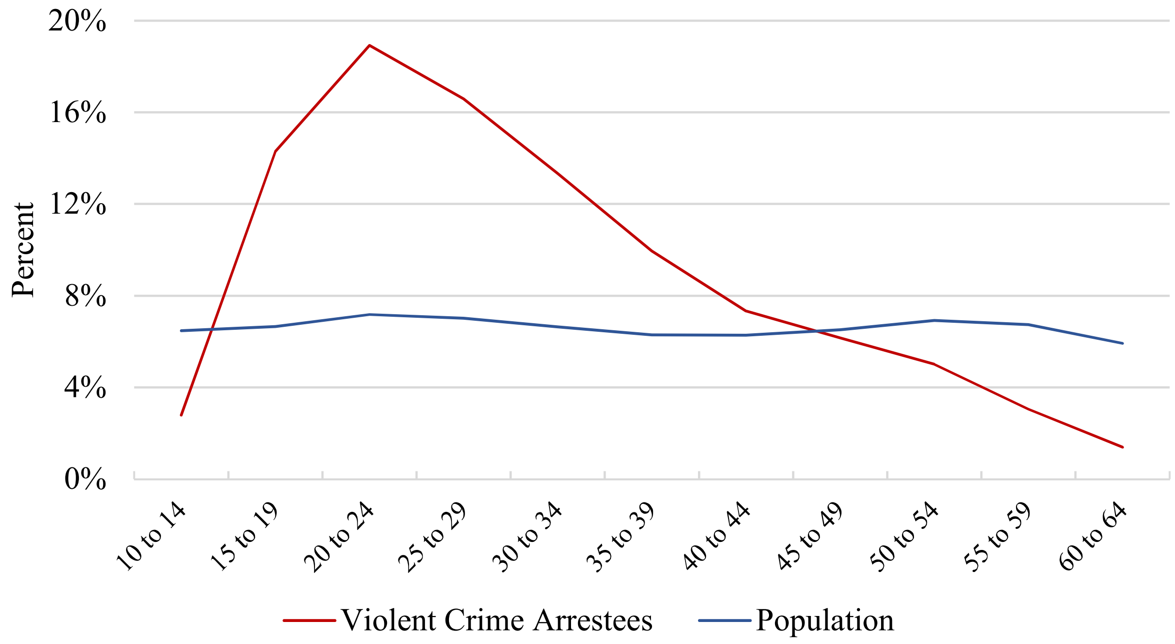

Supplement: S1 Fig — Shown is the age distributions of arrestees for violent crimes in the United States, and of percent of the United States population by age group in 2015. Arrest data are from the Uniform Crime Report of the United States Federal Bureau of Investigation. Population data are from the United Nations World Population Prospects. (PDF) [file pone.0222996.s001.pdf]
