# Supplementary material for: The contribution of age structure to the international homicide decline
Source: PLoS One. 2019 Oct 9;14(10):e0222996. doi: 10.1371/journal.pone.0222996 (PMC6784918; doi:10.1371/journal.pone.0222996)

S3 Fig. Homicide rate and prison rate – United States, 1950 to 2016. Shown is the annual homicide rate per 100,000 population and the percent of the Gross Domestic Product (GDP) in the United States from 1950 to 2015. Homicide data are from the United Nations Office of on Drugs and Crime Homicide Database and the World Health Organization Mortality Database. Data on the prison rate are from the United States Bureau of Justice Statistics.

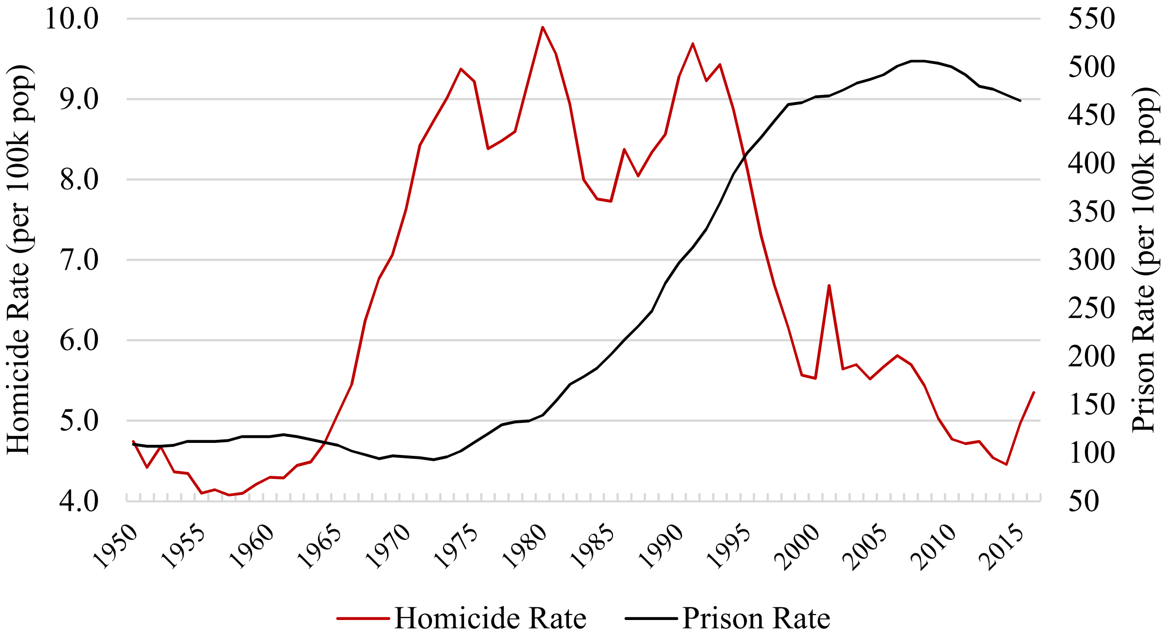

Supplement: S3 Fig — Shown is the annual homicide rate per 100,000 population and the percent of the Gross Domestic Product (GDP) in the United States from 1950 to 2015. Homicide data are from the United Nations Office of on Drugs and Crime Homicide Database and the World Health Organization Mortality Database. Data on the prison rate are from the United States Bureau of Justice Statistics. (PDF) [file pone.0222996.s003.pdf]
