# Supplementary material for: The contribution of age structure to the international homicide decline
Source: PLoS One. 2019 Oct 9;14(10):e0222996. doi: 10.1371/journal.pone.0222996 (PMC6784918; doi:10.1371/journal.pone.0222996)

S5 Fig. World map of homicide rates. The map is an illustration and makes no political statement. Some countries or entities are too small to be visible in the map. This map was created using the website mapchart.net. Reprinted from https://mapchart.net/world.html under a CC BY license, with permission from Minas Giannekas (owner and creator of the map-making website mapchart.net).

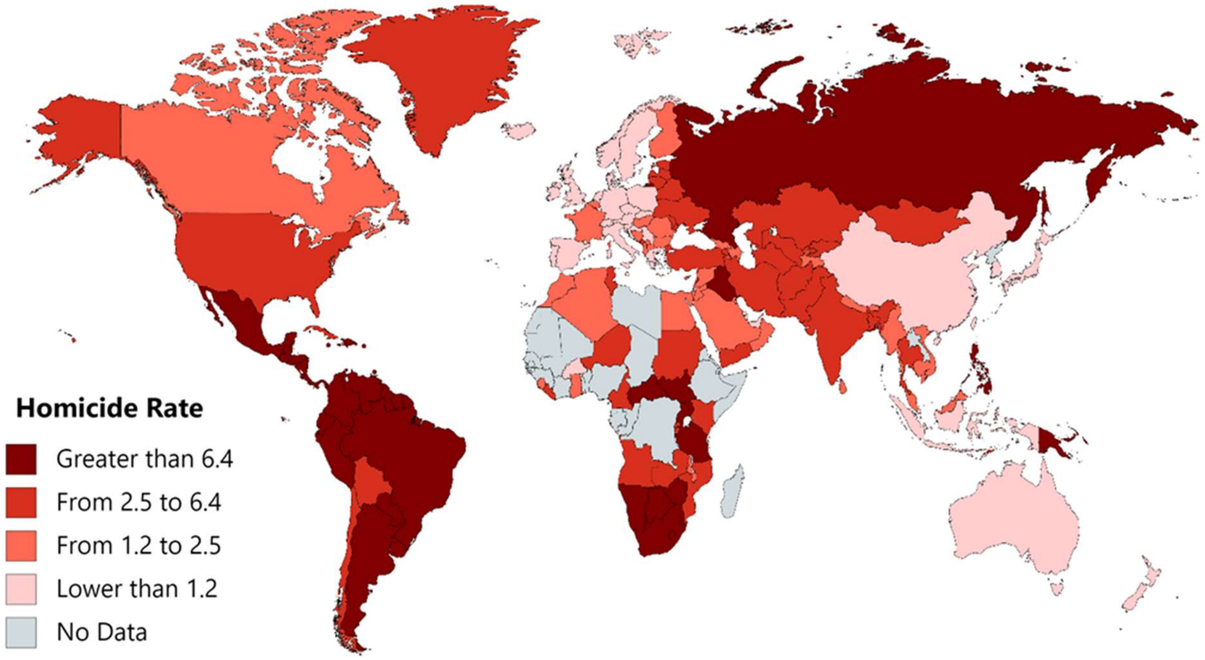

Supplement: S5 Fig — The map is an illustration and makes no political statement. Some countries or entities are too small to be visible in the map. This map was created using the website mapchart.net. Reprinted from https://mapchart.net/world.html under a CC BY license, with permission from Minas Giannekas (owner and creator of the map-making website mapchart.net). (PDF) [file pone.0222996.s005.pdf]
