# Supplementary material for: The contribution of age structure to the international homicide decline
Source: PLoS One. 2019 Oct 9;14(10):e0222996. doi: 10.1371/journal.pone.0222996 (PMC6784918; doi:10.1371/journal.pone.0222996)
Supplement: S6 Fig — The Ratio corresponds to four-year average between the WHO homicide rate and the UN homicide rate between 1990 and 1993. The correlation corresponds to the Pearson correlation between the both rates over all overlapping years in the two series. (PDF) [file pone.0222996.s006.pdf]

S6 Fig. Homicide rate trend by source for countries with combined series. The Ratio corresponds to four-year average between the WHO homicide rate and the UN homicide rate between 1990 and 1993. The correlation corresponds to the Pearson correlation between the both rates over all overlapping years in the two series.

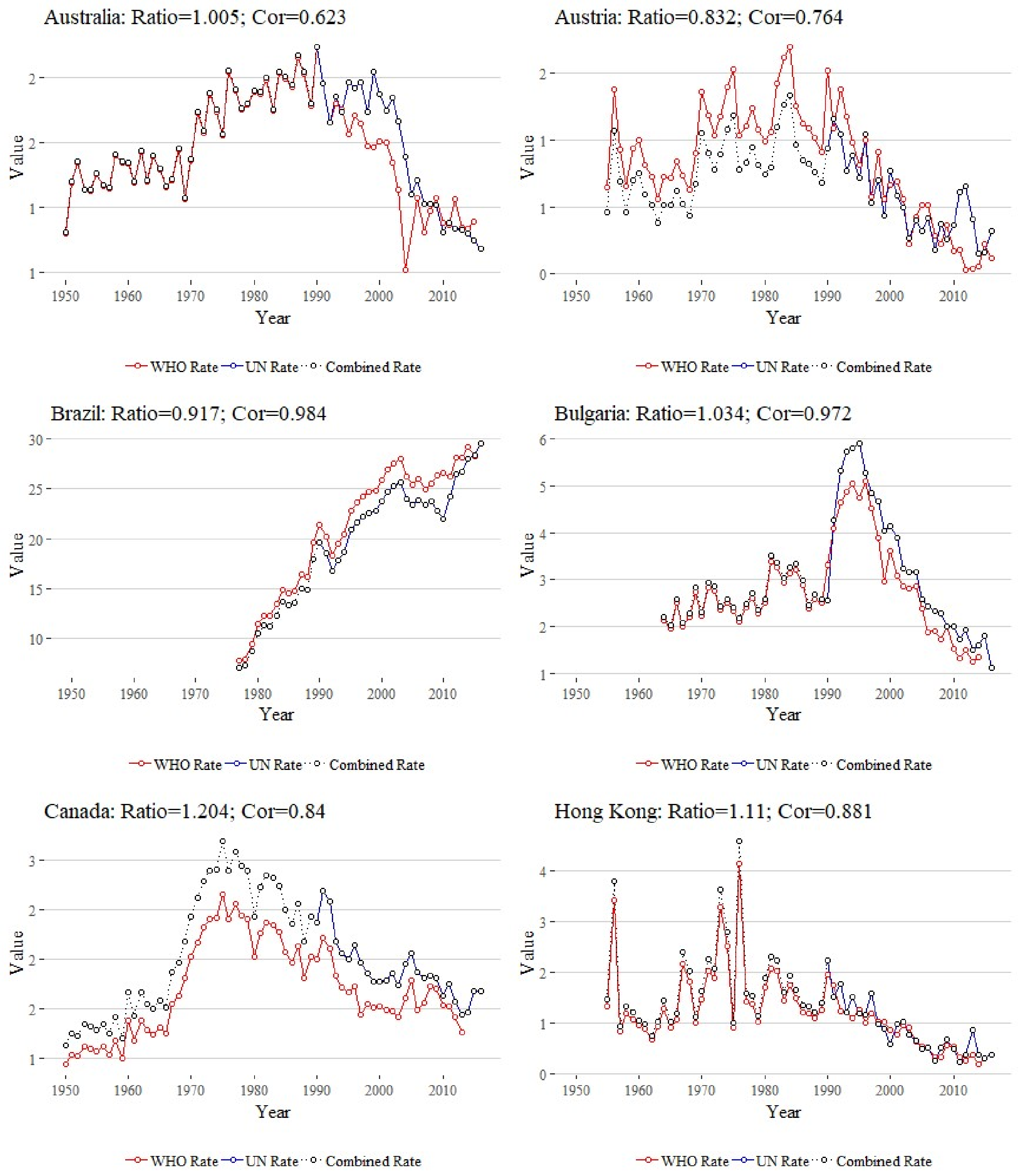

Colombia: Ratio=0.97; Cor=0.978

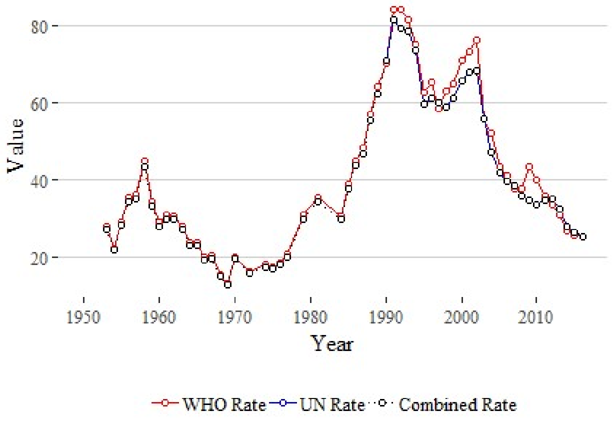

Costa Rica: Ratio=1.003; Cor=0.978

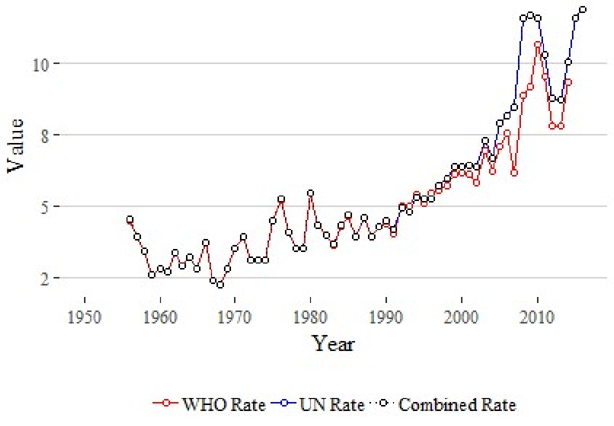

Denmark: Ratio=1.094; Cor=0.64

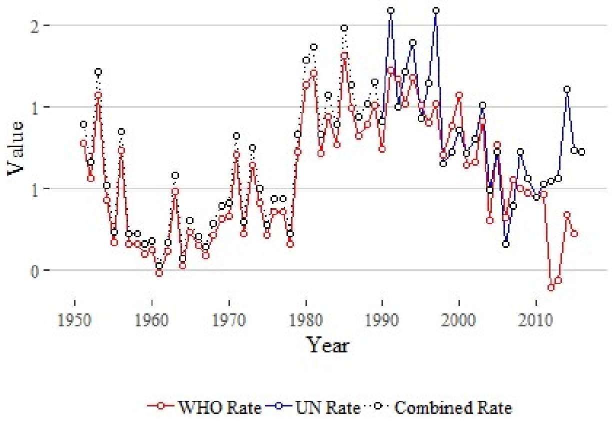

Ecuador: Ratio=0.79; Cor=0.896

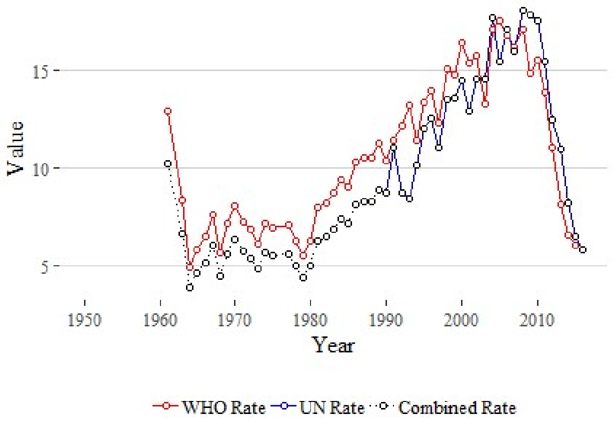

Finland: Ratio=0.909; Cor=0.922

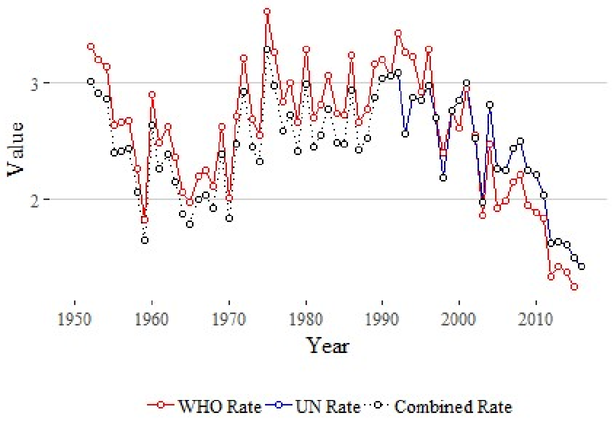

Greece: Ratio=1.028; Cor=0.885

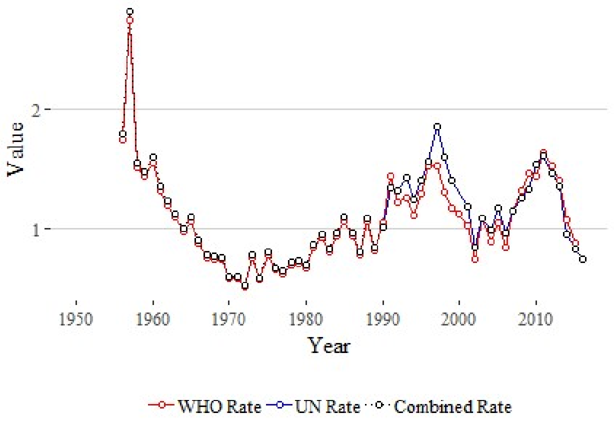

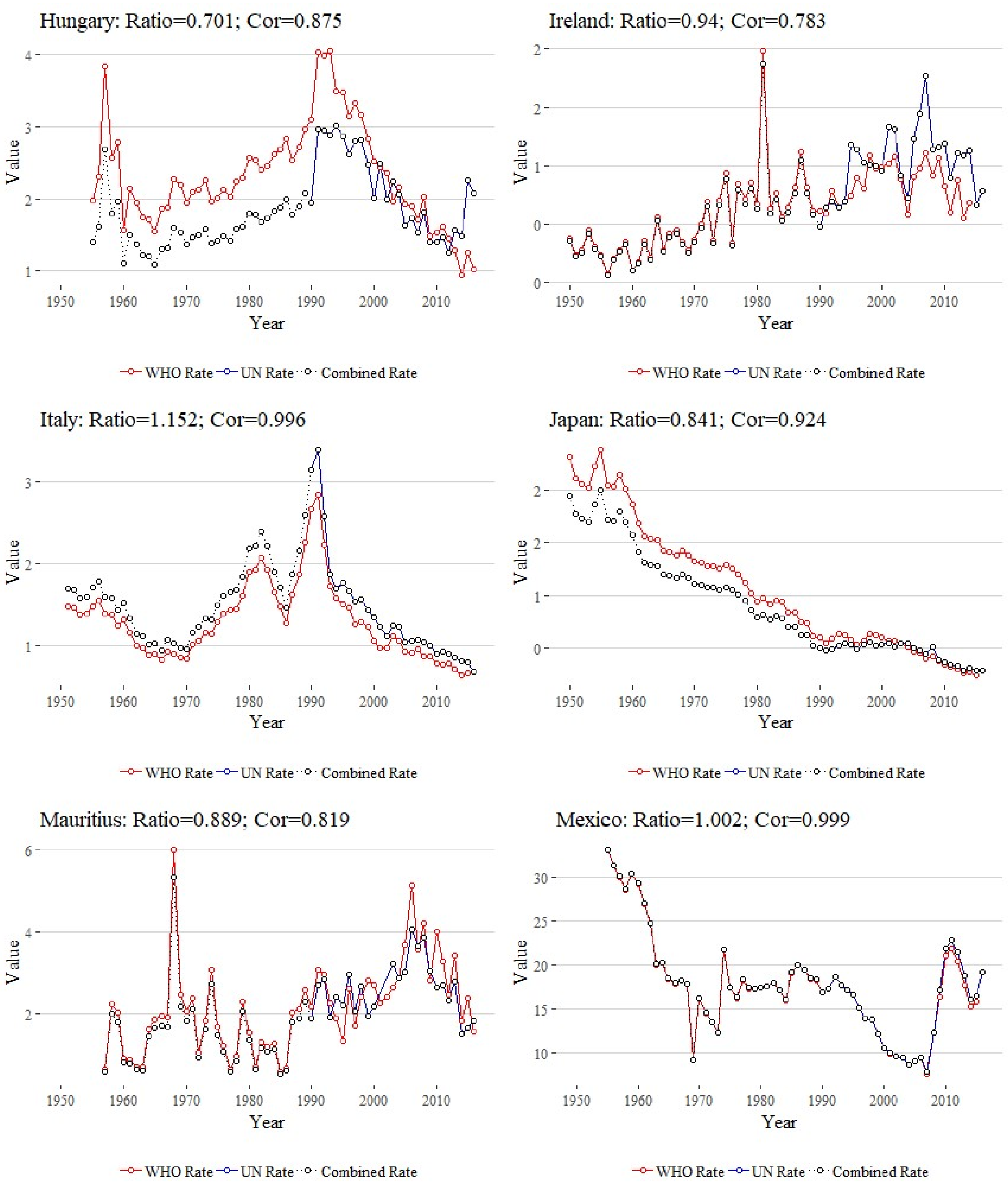
Hungary: Ratio=0.701; Cor=0.875
Value
Year
WHO Rate
UN Rate
Combined Rate
Ireland: Ratio=0.94; Cor=0.783
Value
Year
WHO Rate
UN Rate
Combined Rate
Italy: Ratio=1.152; Cor=0.996
Value
Year
WHO Rate
UN Rate
Combined Rate
Japan: Ratio=0.841; Cor=0.924
Value
Year
WHO Rate
UN Rate
Combined Rate
Mauritius: Ratio=0.889; Cor=0.819
Value
Year
WHO Rate
UN Rate
Combined Rate
Mexico: Ratio=1.002; Cor=0.999
Value
Year
WHO Rate
UN Rate
Combined Rate

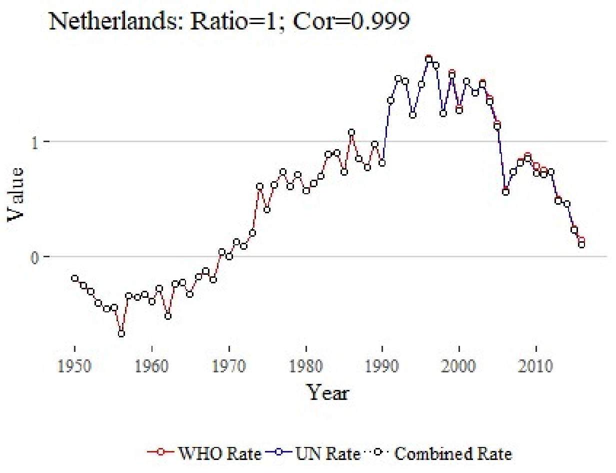
Netherlands: Ratio=1; Cor=0.999
Value
0
1
1950
1960
1970
1980
1990
2000
2010
Year
WHO Rate
UN Rate
Combined Rate

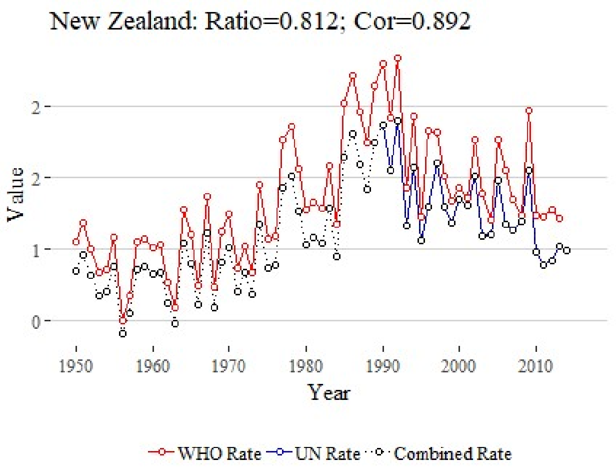
New Zealand: Ratio=0.812; Cor=0.892
Value
0
1
2
2
1950
1960
1970
1980
1990
2000
2010
Year
WHO Rate
UN Rate
Combined Rate

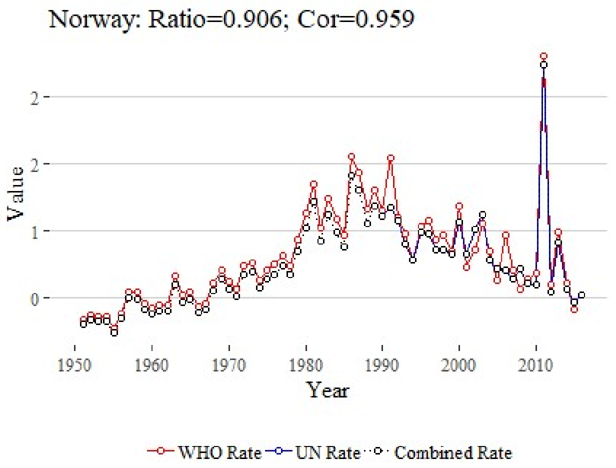
Norway: Ratio=0.906; Cor=0.959
Value
0
1
2
2
1950
1960
1970
1980
1990
2000
2010
Year
WHO Rate
UN Rate
Combined Rate

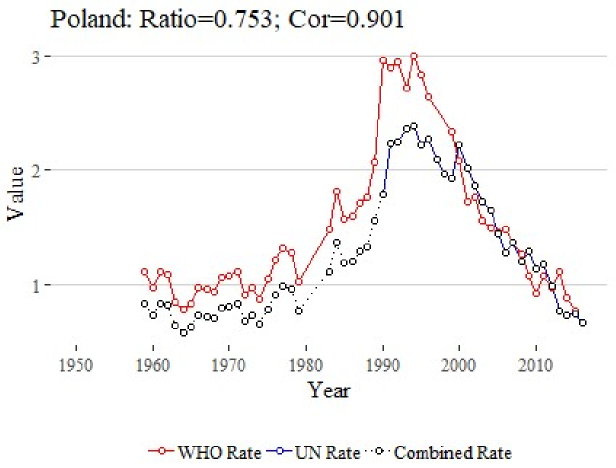
Poland: Ratio=0.753; Cor=0.901
Value
1
2
3
1950
1960
1970
1980
1990
2000
2010
Year
WHO Rate
UN Rate
Combined Rate

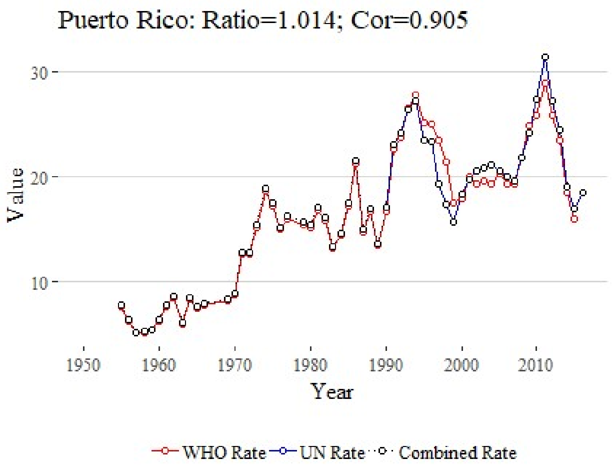
Puerto Rico: Ratio=1.014; Cor=0.905
Value
10
20
30
1950
1960
1970
1980
1990
2000
2010
Year
WHO Rate
UN Rate
Combined Rate

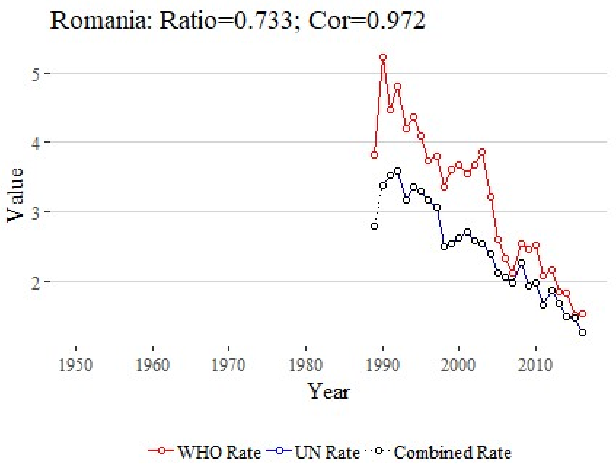
Romania: Ratio=0.733; Cor=0.972
Value
2
3
4
5
1950
1960
1970
1980
1990
2000
2010
Year
WHO Rate
UN Rate
Combined Rate

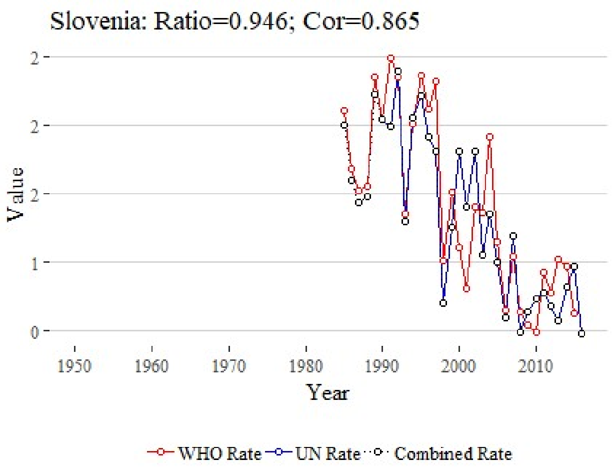
Slovenia: Ratio=0.946; Cor=0.865
Value
Year
1950 1960 1970 1980 1990 2000 2010
WHO Rate UN Rate Combined Rate

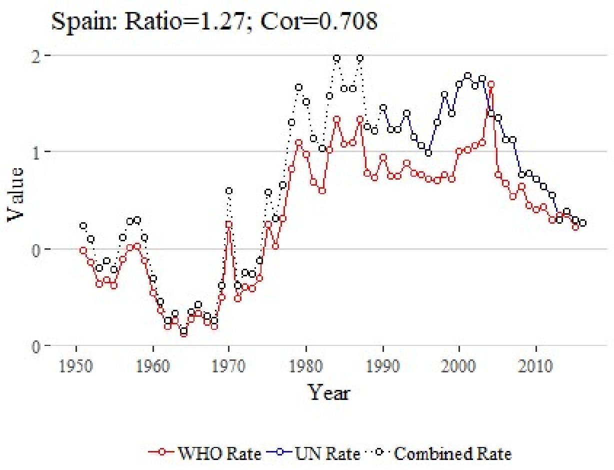
Spain: Ratio=1.27; Cor=0.708
Value
Year
1950 1960 1970 1980 1990 2000 2010
WHO Rate UN Rate Combined Rate

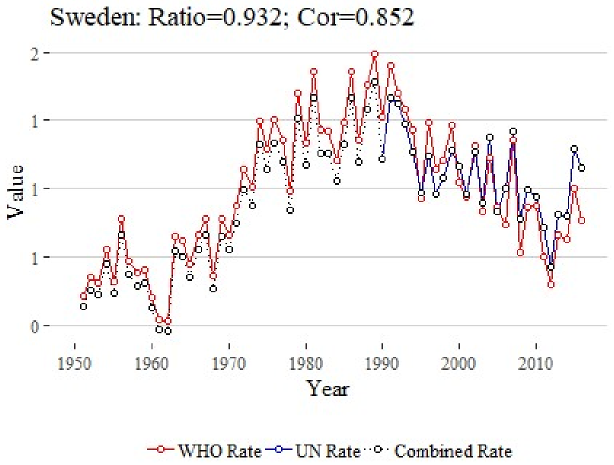
Sweden: Ratio=0.932; Cor=0.852
Value
Year
1950 1960 1970 1980 1990 2000 2010
WHO Rate UN Rate Combined Rate

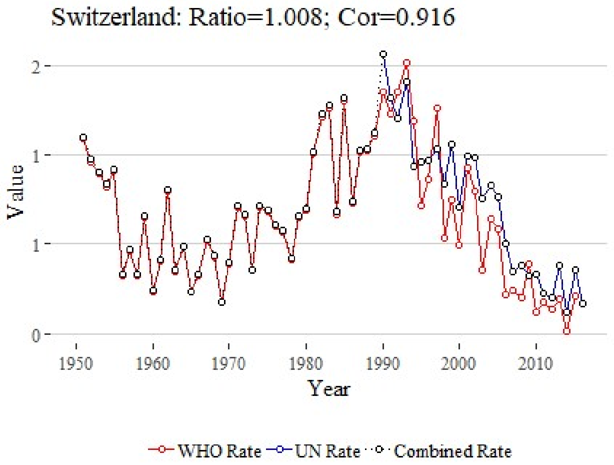
Switzerland: Ratio=1.008; Cor=0.916
Value
Year
1950 1960 1970 1980 1990 2000 2010
WHO Rate UN Rate Combined Rate

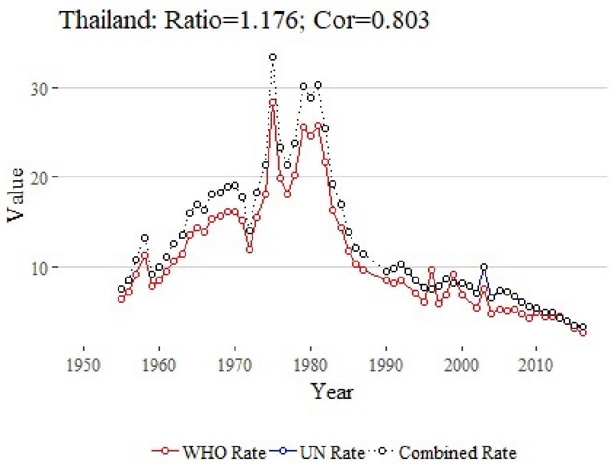
Thailand: Ratio=1.176; Cor=0.803
Value
10 20 30
Year
1950 1960 1970 1980 1990 2000 2010
WHO Rate UN Rate Combined Rate

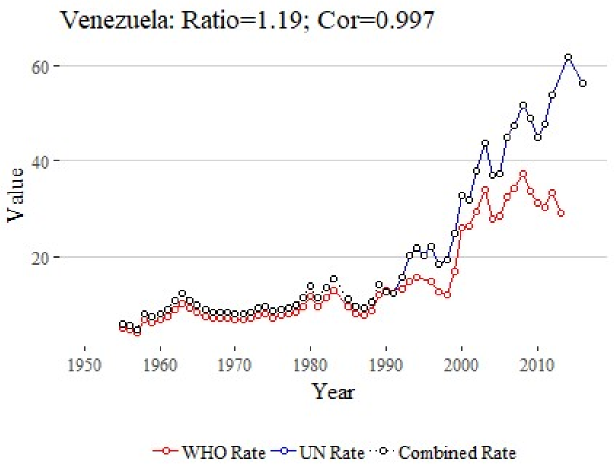
Venezuela: Ratio=1.19; Cor=0.997
Value
20 40 60
Year
1950 1960 1970 1980 1990 2000 2010
WHO Rate UN Rate Combined Rate
