# Supplementary material for: The contribution of age structure to the international homicide decline
Source: PLoS One. 2019 Oct 9;14(10):e0222996. doi: 10.1371/journal.pone.0222996 (PMC6784918; doi:10.1371/journal.pone.0222996)

S7 Fig. Moving average estimate of the homicide trend – Honduras, 1990 to 2015. Shown is the observed and estimated homicide rate for Honduras from 1990 to 2015. The redline and open circles correspond to the estimated rate using an exponentially weighted average. The blackline and closed circles correspond to the observed homicide rate. Homicide data are from the United Nations Office of on Drugs and Crime Homicide Database

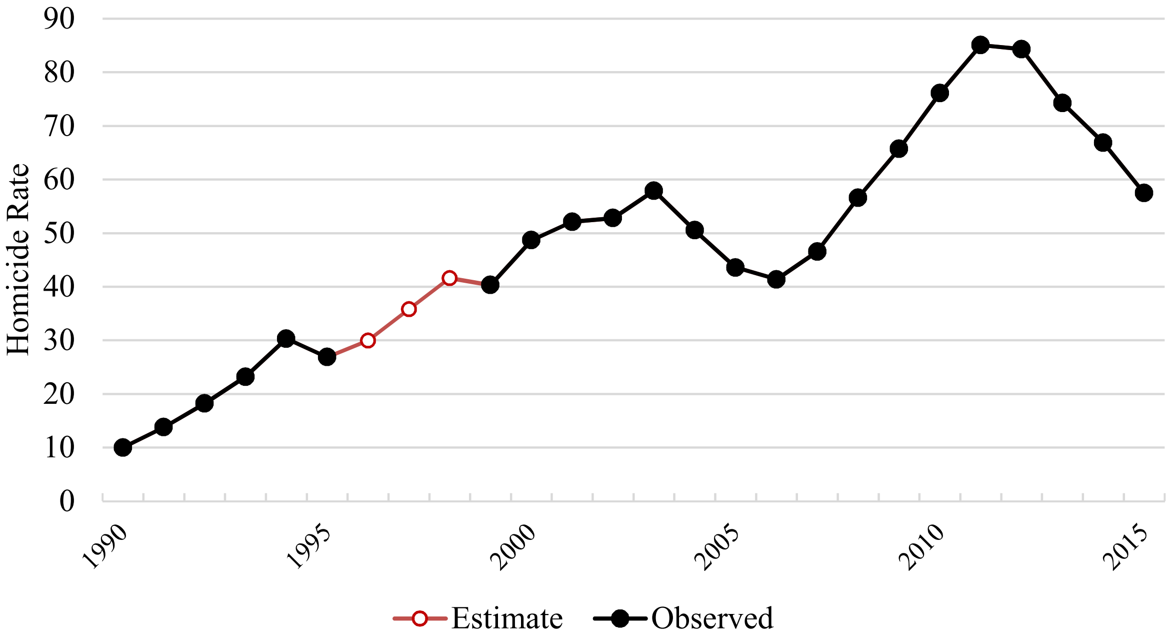

Supplement: S7 Fig — Shown is the observed and estimated homicide rate for Honduras from 1990 to 2015. The redline and open circles correspond to the estimated rate using an exponentially weighted average. The blackline and closed circles correspond to the observed homicide rate. Homicide data are from the United Nations Office of on Drugs and Crime Homicide Database. (PDF) [file pone.0222996.s007.pdf]
