# Supplementary material for: The contribution of age structure to the international homicide decline
Source: PLoS One. 2019 Oct 9;14(10):e0222996. doi: 10.1371/journal.pone.0222996 (PMC6784918; doi:10.1371/journal.pone.0222996)

S8 Fig. Homicide rate and percent of Population 15 to 29 by region – 1990 to 2015. Shown is the homicide rate and the percent of the population 15 and 29 from 1990 and 2015. The percent 15 to 29 is in the secondary axis. Both axis in this figure correspond to the actual values of the homicide rate and of the percent 15 to 29 in each year, and not to the change in these series from 1990. The Pearson correlation between the two series is in parenthesis. Each region is in a part of the figure. Africa is omitted due to lack of data representing the entire region**.** (A) Global Trend. (B) Northern America. (C) Western Europe. (D) Eastern Europe. (E) Asia. (F) Oceania.

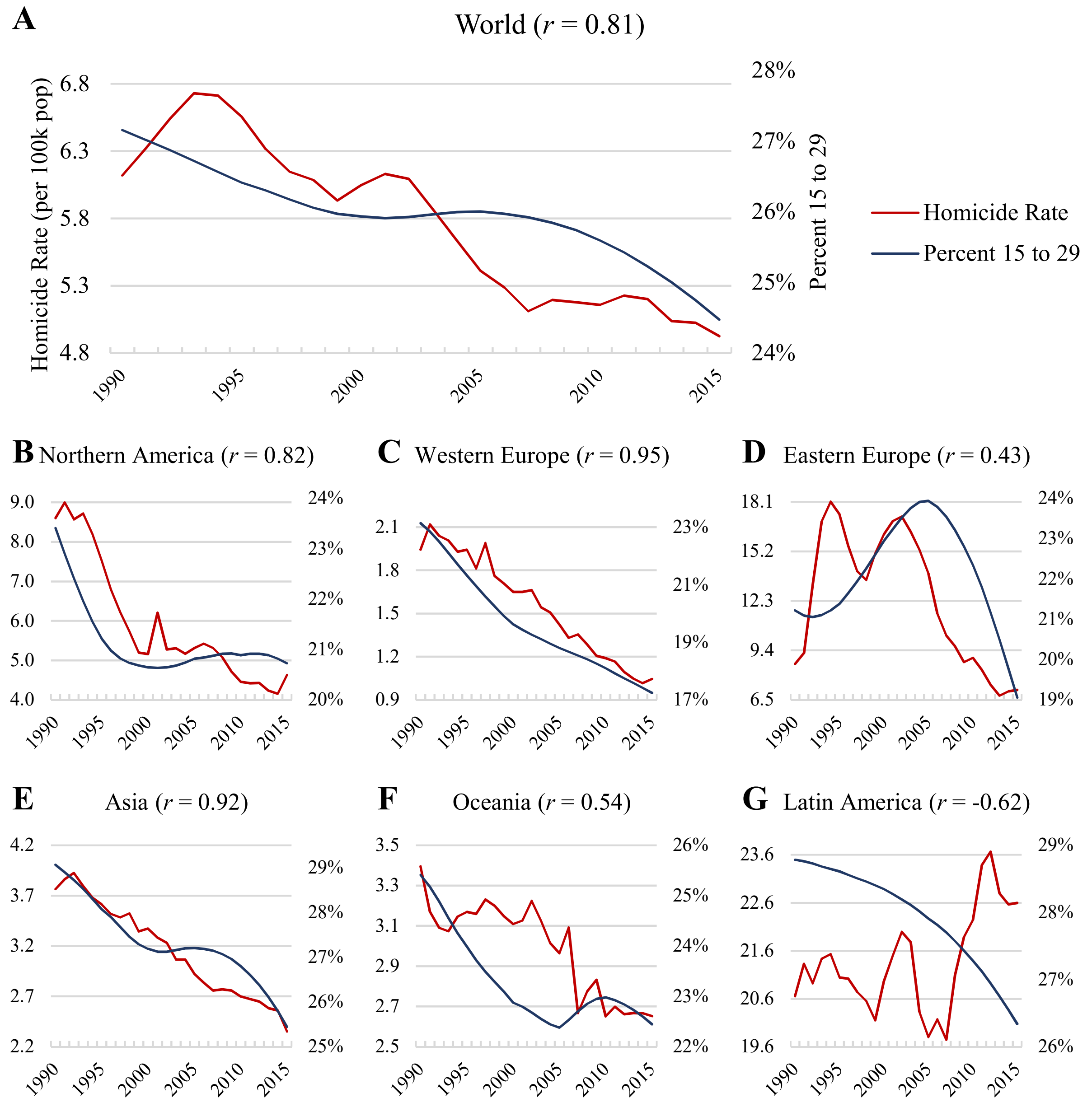

Supplement: S8 Fig — Shown is the homicide rate and the percent of the population 15 and 29 from 1990 and 2015. The percent 15 to 29 is in the secondary axis. Both axis in this figure correspond to the actual values of the homicide rate and of the percent 15 to 29 in each year, and not to the change in these series from 1990. The Pearson correlation between the two series is in parenthesis. Each region is in a part of the figure. Africa is omitted due to lack of data representing the entire region. (A) Global Trend. (B) Northern America. (C) Western Europe. (D) Eastern Europe. (E) Asia. (F) Oceania. (PDF) [file pone.0222996.s008.pdf]
