# Supplementary material for: The contribution of age structure to the international homicide decline
Source: PLoS One. 2019 Oct 9;14(10):e0222996. doi: 10.1371/journal.pone.0222996 (PMC6784918; doi:10.1371/journal.pone.0222996)

S9 Fig. Homicide rate and percent of population 15 to 29 – Low correlations, 1950 to 2016. Shown is the annual homicide rate per 100,000 population and the percent of the population aged 15 to 29 for Brazil, Mexico, and Venezuela from 1950 to 2015. Homicide data are from the United Nations Office of on Drugs and Crime Homicide Database and the World Health Organization Mortality Database. Data on the Percent 15 to 29 are from the United Nations World Population Prospects.

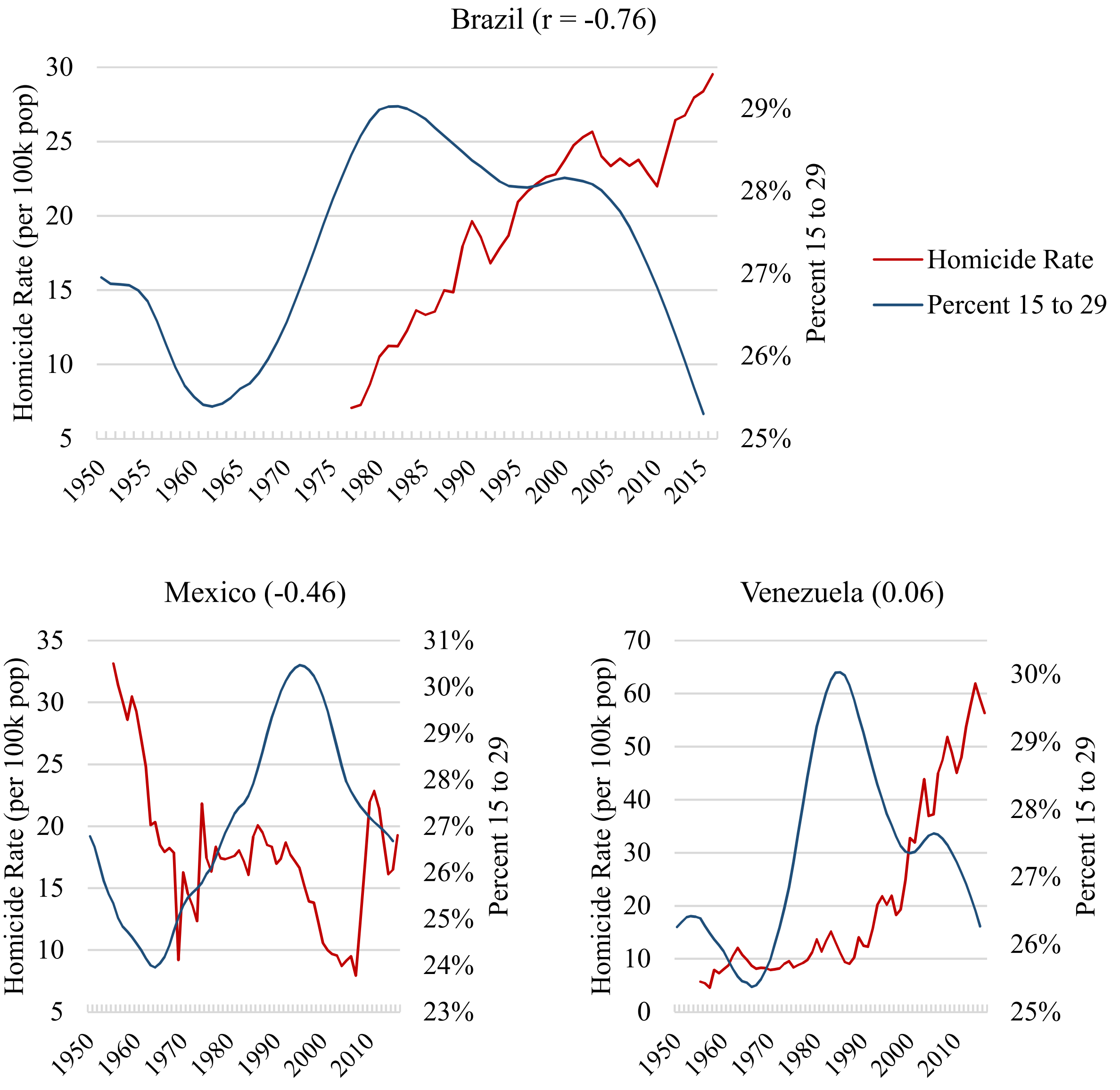

Supplement: S9 Fig — Shown is the annual homicide rate per 100,000 population and the percent of the population aged 15 to 29 for Brazil, Mexico, and Venezuela from 1950 to 2015. Homicide data are from the United Nations Office of on Drugs and Crime Homicide Database and the World Health Organization Mortality Database. Data on the Percent 15 to 29 are from the United Nations World Population Prospects. (PDF) [file pone.0222996.s009.pdf]
