# Supplementary material for: The contribution of age structure to the international homicide decline
Source: PLoS One. 2019 Oct 9;14(10):e0222996. doi: 10.1371/journal.pone.0222996 (PMC6784918; doi:10.1371/journal.pone.0222996)
Supplement: S1 Table — (PDF) [file pone.0222996.s010.pdf]

S1 Table. Considerations for data validation in the United Nations Homicide Statistics.

1. The consistency of the data with a standardized definition of homicides, as mentioned in the International Classification of Crime for Statistical Purposes – the UN specific set of methodological standards for defining and measuring crime and criminal justice outcomes. For instance, if homicide counts excluded non-intentional (e.g. manslaughter) and legal killings (e.g. self-defence);
2. The presence of a clear documentation detailing the source and the methodology associated with collecting the data;
3. The presence of more data observations, and the coherence of each of those observations with each other. Longer series of data from the same source which were robust, and without any artificial breaks were deemed better then single data points. In addition, sources with data on victims by sex, by age group and on other disaggregation which were consistent with the total homicide count was an indicator for quality.
4. If there was a clear indication that homicide data covered information about all homicides in the country. For instance, did data exclude a particular geographical territory, or records from a particular police organization?
5. The specific counting rules used, specifically if only completed homicides were counted, and if the count provided reflected the number of homicide victims, as opposed to cases, offenses or investigations.
6. If the primary source of data constituted an official governmental institution, or an otherwise credible source of country-level data.
7. Each country's government is given the opportunity to revise homicide statistics prior to publication, when they are requested to correct any inaccuracies, or to clarify any remaining issues. Any new data submitted by countries at this stage goes through all the same validation steps as mentioned above, and may still be excluded from publication, if they are not deemed valid.
