# Supplementary material for: The contribution of age structure to the international homicide decline
Source: PLoS One. 2019 Oct 9;14(10):e0222996. doi: 10.1371/journal.pone.0222996 (PMC6784918; doi:10.1371/journal.pone.0222996)
Supplement: S2 Table — (PDF) [file pone.0222996.s011.pdf]

S2 Table. Classification codes for homicides in the WHO Mortality Database by version of the International Classification of Diseases (ICD).

List of ICD revision used	Description	Codes for Homicide
07A	ICD 7th revision, List A (condensed)	A149
07B	ICD 7th revision, List B (condensed)	B050
08A	ICD 8th revision, List A (condensed)	A148
08B	ICD 8th revision, List B (condensed)	B050
09A,09B	ICD 9th revision, Basic Tabulation List (condensed)	B55
09N	ICD 9th revision, Special List of causes (condensed) as reported by some countries of the newly independent States of former USSR	B55, CH17
09C	ICD 9th revision, Special List of causes (condensed) as reported by China	C103
101	ICD10 Mortality Tabulation List 1(condensed)	1102, 1103
103	ICD10 3 (detailed) character list	X85-X99; Y00-Y09
104	ICD10 4 (detailed) character list	X850-X999; Y000-Y099; Y871
10M	ICD10 3 and 4 (detailed) character list	X85-X99; Y00-Y09; X850-X999; Y000-Y099; Y871
UE1	ICD10 special list for Portugal - data for 2004-2005	UE64
