# Supplementary material for: The contribution of age structure to the international homicide decline
Source: PLoS One. 2019 Oct 9;14(10):e0222996. doi: 10.1371/journal.pone.0222996 (PMC6784918; doi:10.1371/journal.pone.0222996)
Supplement: S3 Table — (PDF) [file pone.0222996.s012.pdf]

## S3 Table. List of countries with summary of data availability.

| Region | Country | Total Years | Years | | Homicide Rate | | | |
|---|---|---|---|---|---|---|---|---|
| | | | First | Last | Mean | SD | Min | Max |
| *Long Series Sample (Since 1960)* | | | | | | | | |
| *Africa* | Mauritius | 58 | 1957 | 2016 | 1.97 | 0.99 | 0.53 | 5.34 |
| *Asia* | Hong Kong | 62 | 1955 | 2016 | 1.36 | 0.85 | 0.24 | 4.58 |
| | Japan | 67 | 1950 | 2016 | 0.91 | 0.49 | 0.28 | 2.00 |
| | Thailand | 60 | 1955 | 2016 | 12.85 | 7.38 | 3.24 | 33.45 |
| *Eastern Europe* | Hungary | 62 | 1955 | 2016 | 1.83 | 0.52 | 1.09 | 3.02 |
| | Poland | 55 | 1959 | 2016 | 1.25 | 0.56 | 0.58 | 2.39 |
| *Latin America* | Colombia | 58 | 1953 | 2016 | 39.65 | 18.75 | 13.03 | 81.41 |
| | Costa Rica | 61 | 1956 | 2016 | 5.40 | 2.66 | 2.24 | 11.90 |
| | Mexico | 62 | 1955 | 2016 | 17.83 | 5.78 | 7.93 | 33.14 |
| | Puerto Rico | 59 | 1955 | 2016 | 16.47 | 6.55 | 5.12 | 31.40 |
| | Venezuela | 59 | 1955 | 2016 | 20.13 | 15.95 | 4.56 | 61.91 |
| *Northern America* | Canada | 67 | 1950 | 2016 | 2.02 | 0.56 | 1.13 | 3.19 |
| | United States | 67 | 1950 | 2016 | 6.55 | 1.92 | 4.08 | 9.89 |
| *Oceania* | Australia | 67 | 1950 | 2016 | 1.60 | 0.34 | 0.94 | 2.19 |
| | New Zealand | 65 | 1950 | 2014 | 1.10 | 0.35 | 0.41 | 1.90 |
| *Western Europe* | Austria | 62 | 1955 | 2016 | 0.95 | 0.23 | 0.52 | 1.47 |
| | Denmark | 66 | 1951 | 2016 | 0.96 | 0.32 | 0.43 | 1.67 |
| | Finland | 65 | 1952 | 2016 | 2.44 | 0.45 | 1.42 | 3.29 |
| | Greece | 60 | 1956 | 2016 | 1.11 | 0.40 | 0.53 | 2.81 |
| | Ireland | 67 | 1950 | 2016 | 0.70 | 0.40 | 0.07 | 1.87 |
| | Italy | 66 | 1951 | 2016 | 1.49 | 0.55 | 0.67 | 3.39 |
| | Netherlands | 67 | 1950 | 2016 | 0.76 | 0.33 | 0.17 | 1.36 |
| | Norway | 66 | 1951 | 2016 | 0.74 | 0.34 | 0.24 | 2.24 |
| | Spain | 66 | 1951 | 2016 | 0.83 | 0.41 | 0.08 | 1.49 |
| | Sweden | 66 | 1951 | 2016 | 0.96 | 0.23 | 0.48 | 1.39 |
| | Switzerland | 66 | 1951 | 2016 | 0.96 | 0.28 | 0.50 | 1.65 |
| *High Coverage Sample (Since 1990; includes the above)* | | | | | | | | |
| *Africa* | Algeria | 13 | 2003 | 2015 | 1.10 | 0.41 | 0.61 | 2.01 |
| | Botswana | 10 | 2001 | 2010 | 14.61 | 1.31 | 12.14 | 17.29 |
| | Burkina Faso | 14 | 2002 | 2015 | 0.59 | 0.12 | 0.37 | 0.78 |
| | Burundi | 9 | 2008 | 2016 | 4.82 | 0.81 | 3.86 | 6.02 |
| | Cameroon | 9 | 2000 | 2012 | 4.91 | 1.22 | 3.01 | 6.57 |
| | Egypt | 15 | 1990 | 2012 | 1.04 | 0.87 | 0.37 | 3.15 |
| | Ghana | 11 | 2001 | 2011 | 1.91 | 0.19 | 1.68 | 2.23 |
| | Kenya | 13 | 2004 | 2016 | 4.29 | 1.03 | 2.89 | 5.47 |
| | Lesotho | 13 | 1990 | 2015 | 37.31 | 5.24 | 30.67 | 48.09 |
| | Liberia | 6 | 2007 | 2012 | 3.66 | 0.70 | 2.85 | 4.83 |
| | Malawi | 13 | 2000 | 2012 | 3.99 | 1.99 | 1.53 | 7.70 |
| | Morocco | 24 | 1990 | 2015 | 1.35 | 0.54 | 0.36 | 2.63 |
| | Mozambique | 11 | 2001 | 2011 | 4.57 | 0.93 | 3.40 | 5.92 |
| | Namibia | 14 | 1995 | 2012 | 18.47 | 2.59 | 13.90 | 22.53 |
| | Niger | 2 | 2011 | 2012 | 4.66 | 0.30 | 4.44 | 4.87 |
| | Rwanda | 8 | 2006 | 2015 | 2.82 | 0.72 | 1.48 | 3.77 |
| | Sierra Leone | 10 | 2004 | 2015 | 2.20 | 0.49 | 1.67 | 2.98 |
| | South Africa | 23 | 1994 | 2016 | 42.38 | 11.03 | 29.76 | 63.86 |
| | Sudan | 2 | 2007 | 2008 | 4.92 | 0.34 | 4.68 | 5.16 |
| | Swaziland | 15 | 1990 | 2010 | 16.13 | 2.82 | 10.80 | 19.20 |
| | Tanzania | 11 | 1995 | 2015 | 7.66 | 0.58 | 6.95 | 8.72 |
| | Tunisia | 8 | 2004 | 2012 | 2.56 | 0.31 | 1.95 | 3.05 |
| | Uganda | 15 | 1995 | 2014 | 9.28 | 1.19 | 7.43 | 11.52 |

| Region | Country | Total Years | Years | | Homicide Rate | | | |
|---|---|---|---|---|---|---|---|---|
| | | | First | Last | Mean | SD | Min | Max |
| | Zambia | 16 | 1990 | 2015 | 7.16 | 1.70 | 5.28 | 10.47 |
| | Zimbabwe | 9 | 1990 | 2012 | 9.20 | 2.89 | 5.05 | 12.98 |
| *Asia* | Afghanistan | 4 | 2009 | 2012 | 4.47 | 1.29 | 3.41 | 6.35 |
| | Armenia | 27 | 1990 | 2016 | 3.42 | 1.68 | 1.95 | 8.86 |
| | Azerbaijan | 24 | 1990 | 2016 | 3.48 | 1.66 | 2.14 | 7.55 |
| | Bangladesh | 16 | 2000 | 2015 | 2.66 | 0.13 | 2.50 | 2.87 |
| | Cambodia | 20 | 1992 | 2011 | 3.70 | 1.29 | 1.84 | 6.84 |
| | China | 22 | 1995 | 2016 | 1.46 | 0.58 | 0.62 | 2.21 |
| | Cyprus | 27 | 1990 | 2016 | 1.00 | 0.47 | 0.13 | 1.95 |
| | Georgia | 23 | 1990 | 2016 | 6.86 | 3.48 | 0.99 | 16.87 |
| | India | 27 | 1990 | 2016 | 4.50 | 0.88 | 3.22 | 6.15 |
| | Indonesia | 14 | 1998 | 2016 | 0.65 | 0.19 | 0.44 | 1.04 |
| | Iran | 5 | 2003 | 2014 | 2.72 | 0.22 | 2.47 | 3.01 |
| | Iraq | 11 | 1990 | 2013 | 8.42 | 2.72 | 5.88 | 15.79 |
| | Israel | 25 | 1990 | 2015 | 2.44 | 0.62 | 1.36 | 3.64 |
| | Jordan | 20 | 1990 | 2016 | 2.08 | 1.05 | 0.77 | 4.48 |
| | Kazakhstan | 23 | 1990 | 2015 | 11.92 | 3.87 | 4.81 | 16.59 |
| | Kyrgyzstan | 27 | 1990 | 2016 | 8.90 | 3.31 | 3.62 | 19.77 |
| | Lebanon | 9 | 2008 | 2016 | 4.00 | 0.35 | 3.47 | 4.47 |
| | Malaysia | 24 | 1990 | 2013 | 2.15 | 0.28 | 1.68 | 2.84 |
| | Mongolia | 14 | 2003 | 2016 | 9.68 | 3.23 | 5.66 | 15.75 |
| | Myanmar | 19 | 1990 | 2016 | 2.18 | 0.89 | 1.39 | 5.04 |
| | Nepal | 20 | 1990 | 2016 | 2.79 | 0.55 | 1.89 | 3.70 |
| | Pakistan | 22 | 1990 | 2016 | 6.86 | 0.89 | 4.41 | 7.96 |
| | Palestine | 17 | 1995 | 2016 | 1.50 | 1.03 | 0.54 | 4.38 |
| | Philippines | 24 | 1990 | 2016 | 9.18 | 2.25 | 6.41 | 14.98 |
| | Qatar | 20 | 1990 | 2014 | 0.60 | 0.28 | 0.17 | 1.21 |
| | Singapore | 27 | 1990 | 2016 | 0.77 | 0.49 | 0.21 | 1.76 |
| | South Korea | 27 | 1990 | 2016 | 0.74 | 0.16 | 0.48 | 1.08 |
| | Sri Lanka | 18 | 1990 | 2016 | 6.53 | 3.27 | 2.35 | 11.45 |
| | Tajikistan | 22 | 1990 | 2011 | 5.40 | 5.08 | 1.30 | 23.31 |
| | Timor-Leste | 8 | 2004 | 2015 | 3.87 | 1.11 | 2.31 | 5.82 |
| | Turkey | 10 | 2003 | 2012 | 4.58 | 0.38 | 4.17 | 5.18 |
| | Turkmenistan | 17 | 1990 | 2006 | 6.61 | 1.59 | 4.22 | 9.01 |
| | Uzbekistan | 19 | 1990 | 2008 | 4.33 | 0.83 | 2.99 | 5.50 |
| | Viet Nam | 11 | 2001 | 2011 | 1.33 | 0.11 | 1.23 | 1.52 |
| | Yemen | 14 | 1998 | 2013 | 4.62 | 1.11 | 3.23 | 6.66 |
| *Eastern Europe* | Belarus | 25 | 1990 | 2014 | 7.22 | 2.48 | 3.51 | 10.33 |
| | Bulgaria | 53 | 1964 | 2016 | 2.99 | 1.14 | 1.14 | 5.90 |
| | Czechia | 23 | 1994 | 2016 | 1.28 | 0.40 | 0.61 | 2.00 |
| | Moldova | 25 | 1990 | 2014 | 7.62 | 2.06 | 3.19 | 11.40 |
| | Romania | 28 | 1989 | 2016 | 2.46 | 0.68 | 1.25 | 3.59 |
| | Russia | 24 | 1990 | 2016 | 21.97 | 7.25 | 10.82 | 32.25 |
| | Slovakia | 26 | 1990 | 2016 | 1.90 | 0.55 | 0.81 | 2.61 |
| | Ukraine | 23 | 1990 | 2014 | 7.15 | 1.73 | 4.34 | 10.03 |
| *Latin America* | Argentina | 3 | 2014 | 2016 | 6.66 | 0.79 | 5.94 | 7.51 |
| | Bolivia | 12 | 2005 | 2016 | 8.59 | 2.64 | 5.19 | 12.83 |
| | Brazil | 40 | 1977 | 2016 | 19.60 | 6.17 | 7.07 | 29.53 |
| | Chile | 13 | 2003 | 2016 | 3.39 | 0.36 | 2.51 | 3.74 |
| | Dominican Rep | 25 | 1991 | 2016 | 17.77 | 5.40 | 10.81 | 25.92 |
| | Ecuador | 54 | 1961 | 2016 | 9.52 | 4.28 | 3.94 | 18.04 |
| | El Salvador | 23 | 1994 | 2016 | 75.91 | 29.32 | 40.20 | 142.16 |
| | Guatemala | 25 | 1992 | 2016 | 33.97 | 6.26 | 23.32 | 45.39 |
| | Haiti | 10 | 2007 | 2016 | 8.08 | 2.07 | 5.09 | 10.04 |

| Region | Country | Total Years | Years | | Homicide Rate | | | |
|---|---|---|---|---|---|---|---|---|
| | | | First | Last | Mean | SD | Min | Max |
| | Honduras | 24 | 1990 | 2016 | 49.13 | 21.05 | 10.00 | 85.06 |
| | Jamaica | 27 | 1990 | 2016 | 40.11 | 11.35 | 22.44 | 60.99 |
| | Nicaragua | 27 | 1990 | 2016 | 12.91 | 2.85 | 7.37 | 19.07 |
| | Panama | 27 | 1990 | 2016 | 11.69 | 2.62 | 6.52 | 17.27 |
| | Paraguay | 16 | 2000 | 2015 | 15.02 | 5.59 | 8.82 | 24.91 |
| | Peru | 6 | 2011 | 2016 | 6.68 | 0.75 | 5.43 | 7.67 |
| | Trinidad & Tobago | 16 | 2000 | 2015 | 26.38 | 9.37 | 9.46 | 41.59 |
| | Uruguay | 25 | 1990 | 2016 | 6.66 | 0.80 | 5.71 | 8.54 |
| *Oceania* | Papua New Guinea | 5 | 1998 | 2007 | 8.68 | 0.74 | 7.85 | 9.49 |
| *Western Europe* | Albania | 25 | 1992 | 2016 | 7.41 | 8.63 | 2.26 | 43.13 |
| | Belgium | 24 | 1990 | 2015 | 1.81 | 0.53 | 1.02 | 3.08 |
| | Bosnia & Herzegovina | 17 | 1990 | 2016 | 1.76 | 0.37 | 1.28 | 2.63 |
| | Croatia | 27 | 1990 | 2016 | 2.44 | 2.01 | 0.85 | 9.18 |
| | Estonia | 26 | 1990 | 2015 | 9.46 | 4.87 | 3.11 | 20.73 |
| | France | 27 | 1990 | 2016 | 1.73 | 0.42 | 1.22 | 2.63 |
| | Germany | 27 | 1990 | 2016 | 1.19 | 0.30 | 0.81 | 1.74 |
| | Latvia | 24 | 1992 | 2015 | 7.54 | 3.67 | 2.45 | 14.98 |
| | Lithuania | 27 | 1990 | 2016 | 8.67 | 2.39 | 4.87 | 13.84 |
| | Macedonia | 17 | 1998 | 2014 | 2.08 | 0.59 | 1.06 | 3.41 |
| | Portugal | 27 | 1990 | 2016 | 1.22 | 0.24 | 0.64 | 1.74 |
| | Serbia | 17 | 2000 | 2016 | 1.70 | 0.39 | 1.17 | 2.58 |
| | Slovenia | 32 | 1985 | 2016 | 1.34 | 0.59 | 0.48 | 2.39 |
| | United Kingdom | 27 | 1990 | 2016 | 1.36 | 0.25 | 0.91 | 1.87 |
