# Supplementary material for: The contribution of age structure to the international homicide decline
Source: PLoS One. 2019 Oct 9;14(10):e0222996. doi: 10.1371/journal.pone.0222996 (PMC6784918; doi:10.1371/journal.pone.0222996)
Supplement: S4 Table — (PDF) [file pone.0222996.s013.pdf]

S4 Table. List of countries absent from the analytical samples.

Region	Country	Total Years	Years		Homicide Rate			
			First	Last	Mean	SD	Min	Max
Small States (bellow 1 million population)								
Africa	Cabo Verde	14	2003	2016	8.34	2.66	3.26	12.35
	Mayotte	3	2007	2009	7.56	6.00	2.55	14.20
	Réunion	6	2004	2009	2.58	0.52	1.82	3.16
	Sao Tome & Principe	11	1990	2011	5.39	2.60	2.45	10.10
	Seychelles	12	2004	2016	11.72	4.76	4.33	19.40
	St Helena	16	1994	2009	1.42	5.67	0.00	22.69
Asia	Bhutan	26	1990	2016	2.65	1.09	0.80	5.08
	Brunei Darussalam	16	1996	2013	0.99	0.77	0.25	2.95
	Macao	26	1991	2016	2.26	2.37	0.00	8.84
	Maldives	13	1990	2013	1.52	0.78	0.45	2.72
Latin America	Anguilla	25	1990	2014	9.89	11.77	0.00	38.75
	Antigua & Barbuda	20	1990	2012	7.59	5.14	1.29	18.60
	Aruba	17	1990	2014	3.87	1.85	0.00	7.47
	Bahamas	25	1990	2016	21.27	7.55	10.72	37.74
	Barbados	24	1990	2015	9.07	1.80	6.42	13.06
	Belize	17	2000	2016	31.82	6.31	16.58	43.06
	British Virgin Isl	13	1990	2006	5.93	5.37	0.00	17.75
	Cayman Isl	23	1990	2014	6.49	4.00	0.00	16.21
	Curaçao	7	2001	2007	22.35	9.90	7.68	33.59
	Dominica	14	1998	2011	10.61	5.12	1.44	21.00
	French Guiana	6	2004	2009	18.67	6.54	12.92	29.58
	Grenada	17	2000	2016	9.40	3.56	3.81	15.39
	Guadeloupe	6	2004	2009	6.38	1.07	5.23	8.01
	Guyana	27	1990	2016	16.84	4.36	10.09	27.80
	Martinique	6	2004	2009	4.70	1.12	2.78	5.79
	Montserrat	19	1990	2012	7.09	9.03	0.00	20.93
	St Kitts & Nevis	18	1995	2012	25.71	16.89	6.61	65.38
	St Lucia	25	1990	2014	16.83	7.39	5.57	26.46
	St Vincent & the Grenadines	24	1990	2016	19.30	7.20	8.33	36.68
	Suriname	9	2000	2008	11.96	2.75	8.35	16.54
	Turks & Caicos Islands	20	1990	2014	5.06	4.71	0.00	13.70
	Virgin Islands (US)	16	1997	2012	35.76	9.84	21.15	52.76
Northern America	Bermuda	26	1990	2016	5.47	3.71	0.00	12.96
	Greenland	25	1992	2016	15.12	6.88	1.77	30.18
	St Pierre & Miquelon	4	2006	2009	7.97	9.20	0.00	15.95
Oceania	American Samoa	16	2001	2016	6.13	2.87	1.72	11.92
	Cook Islands	1	2012	2012	3.49		3.49	3.49
	Fiji	20	1990	2014	2.69	0.80	1.64	4.57
	French Polynesia	4	2006	2009	1.91	1.39	0.38	3.42
	Guam	12	2000	2011	3.21	2.17	0.63	6.93
	Kiribati	16	1991	2012	2.47	3.39	0.00	10.51
	Marshall Islands	3	1991	1994	6.11	3.64	3.95	10.31
	New Caledonia	4	2006	2009	4.03	0.86	3.23	5.00
	Samoa	3	2009	2013	6.80	3.16	3.15	8.66
	Solomon Islands	5	2004	2008	4.74	0.71	3.77	5.53
	Tonga	16	1995	2012	3.48	2.49	0.95	7.87
	Tuvalu	11	2002	2012	3.41	6.31	0.00	18.65
Western Europe	Andorra	10	2004	2015	0.37	0.60	0.00	1.31
	Channel Islands	4	2005	2010	0.64	0.53	0.00	1.29
	Gibraltar	2	2009	2010	1.51	2.13	0.00	3.01

Region	Country	Total Years	Years		Homicide Rate			
			First	Last	Mean	SD	Min	Max
	Holy See	7	2009	2015	0.00	0.00	0.00	0.00
	Iceland	23	1994	2016	0.54	0.48	0.00	1.78
	Isle of Man	6	2011	2016	1.83	1.27	0.00	3.63
	Liechtenstein	23	1994	2016	0.62	1.20	0.00	3.04
	Luxembourg	21	1994	2014	1.04	0.54	0.00	2.04
	Malta	23	1990	2015	1.25	0.66	0.00	2.85
	Monaco	12	2001	2015	1.00	1.47	0.00	3.09
	Montenegro	15	2002	2016	3.14	0.86	1.59	4.46
	San Marino	17	1995	2011	0.21	0.86	0.00	3.56
Missing Data on control variables								
Africa	Angola	2	2011	2012	4.60	0.35	4.36	4.85
	Central African Rep	1	2016	2016	19.76		19.76	19.76
	South Sudan	1	2012	2012	13.90		13.90	13.90
Asia	Bahrain	17	1995	2014	0.75	0.34	0.31	1.38
	Kuwait	13	1996	2012	1.70	0.39	0.98	2.36
	Oman	13	2002	2014	1.74	1.01	0.66	4.68
	Saudi Arabia	10	1999	2015	1.13	0.20	0.83	1.50
	Syrian Arab Rep	14	1997	2010	2.28	0.20	1.95	2.71
	Taiwan	9	2001	2015	0.87	0.21	0.72	1.39
	United Arab Emirates	11	2003	2016	0.81	0.22	0.59	1.22
Latin America	Cuba	25	1992	2016	5.85	1.03	4.46	8.30
Western Europe	Kosovo	9	2008	2016	3.64	1.85	1.60	6.47
Countries without homicide data								
Africa	Benin	0						
	Chad	0						
	Comoros	0						
	Congo	0						
	Côte d'Ivoire	0						
	Djibouti	0						
	DR Congo	0						
	Equatorial Guinea	0						
	Eritrea	0						
	Ethiopia	0						
	Gabon	0						
	Gambia	0						
	Guinea	0						
	Guinea-Bissau	0						
	Libya	0						
	Madagascar	0						
	Mali	0						
	Mauritania	0						
	Nigeria	0						
	Senegal	0						
	Somalia	0						
	Togo	0						
	Western Sahara	0						
Asia	Lao	0						
	North Korea	0						
Latin America	Caribbean Netherlands	0						
	Falkland Islands	0						
	Sint Maarten (Dutch)	0						
Oceania	Micronesia	0						
	Nauru	0						
	Niue	0						

Region	Country	Total Years	Years		Homicide Rate			
			First	Last	Mean	SD	Min	Max
	Northern Mariana Islands	0						
	Palau	0						
	Tokelau	0						
	Vanuatu	0						
	Wallis & Futuna Islands	0						
Western Europe	Faroe Islands	0						
