# Supplementary material for: The contribution of age structure to the international homicide decline
Source: PLoS One. 2019 Oct 9;14(10):e0222996. doi: 10.1371/journal.pone.0222996 (PMC6784918; doi:10.1371/journal.pone.0222996)
Supplement: S5 Table — Shown is the total sample size (N), mean, standard deviation (SD), interquartile range, minimum, maximum values, and year range of available data for all variables included in the High Coverage Sample and Long Series Sample. (PDF) [file pone.0222996.s014.pdf]

S5 Table. Descriptive statistics by sample. Shown is the total sample size (N), mean, standard deviation (SD), interquartile range, minimum, maximum values, and year range of available data for all variables included in the High Coverage Sample and Long Series Sample.

Statistic	N	Mean	SD	Min	Pctl(25)	Pctl(75)	Max	Earliest	Latest
High Coverage Sample (Since 1990)									
Homicide Rate	2,283	8.12	13.26	0.13	1.36	8.76	142.16	1990	2015
Percent 15 to 29	2,283	24.54	4.00	14.68	21.15	27.80	35.31	1990	2015
Percent Male	2,283	49.34	2.00	45.79	48.66	49.90	76.07	1990	2015
Gini Index	2,283	37.21	8.67	19.90	30.75	43.10	63.00	1990	2015
GDP per Cap (USD 1k)	2,283	15.49	18.28	0.23	2.36	24.48	91.41	1990	2015
Percent Urban	2,283	61.11	21.24	8.85	47.14	76.58	100.00	1990	2015
Long Series Sample (Since 1960)									
Homicide Rate	1,136	6.26	11.09	0.24	1.03	6.27	81.41	1960	2015
Percent 15 to 29	1,136	23.42	3.64	14.68	20.71	26.05	32.79	1960	2015
Percent Male	1,136	49.40	0.76	46.22	48.99	49.84	52.19	1960	2015
Gini Index	1,136	33.96	8.45	20.20	27.40	40.60	53.00	1960	2015
GDP per Cap (USD 1k)	1,136	27.18	18.76	0.61	11.71	38.33	91.41	1960	2015
Percent Urban	1,136	71.89	15.41	19.89	65.44	83.02	100.00	1960	2015
