# Supplementary material for: The contribution of age structure to the international homicide decline
Source: PLoS One. 2019 Oct 9;14(10):e0222996. doi: 10.1371/journal.pone.0222996 (PMC6784918; doi:10.1371/journal.pone.0222996)
Supplement: S6 Table — Shown is the personal correlation coefficient and variance inflation factor (VIF) for all variables in the High Coverage Sample and Long Series Sample. The VIFs are is based on a fixed effects model predicting the Natural Log of Homicide Rate, controlling for all other variables in the matrix, for each sample. (PDF) [file pone.0222996.s015.pdf]

S6 Table. Correlation matrix per sample with variance inflation factor. Shown is the personal correlation coefficient and variance inflation factor (VIF) for all variables in the High Coverage Sample and Long Series Sample. The VIFs are is based on a fixed effects model predicting the Natural Log of Homicide Rate, controlling for all other variables in the matrix, for each sample.

High Coverage Sample (Since 1990)	(1)	(2)	(3)	(4)	(5)	(6)	VIF
(1) Homicide Rate	1.00						
(2) Percent 15 to 29	0.34	1.00					3.10
(3) Percent Male	-0.08	0.32	1.00				1.46
(4) Gini Index	0.53	0.64	0.13	1.00			1.73
(5) GDP per Cap (USD 1k)	-0.28	-0.65	0.14	-0.50	1.00		2.73
(6) Percent Urban	-0.06	-0.50	0.01	-0.31	0.61	1.00	1.65
Long Series Sample (Since 1960)	(1)	(2)	(3)	(4)	(5)	(6)	VIF
(1) Homicide Rate	1.00						
(2) Percent 15 to 29	0.46	1.00					2.27
(3) Percent Male	0.10	0.43	1.00				1.26
(4) Gini Index	0.65	0.53	0.19	1.00			1.70
(5) GDP per Cap (USD 1k)	-0.41	-0.67	-0.17	-0.62	1.00		2.50
(6) Percent Urban	-0.06	-0.32	-0.11	-0.22	0.43	1.00	1.25
