# Supplementary material for: The contribution of age structure to the international homicide decline
Source: PLoS One. 2019 Oct 9;14(10):e0222996. doi: 10.1371/journal.pone.0222996 (PMC6784918; doi:10.1371/journal.pone.0222996)
Supplement: S8 Table — Shown are the coefficients from the quantile regression model with fixed effects for the Long Series Sample. Coefficients are exponentiated and correspond to the average proportional change in the homicide rate from a one-unit increase in the corresponding independent variable. In parenthesis are standard errors clustered by country. ***p < 0.001; **p < 0.01; *p < 0.05. (PDF) [file pone.0222996.s017.pdf]

S8 Table. Quantile regression with fixed effects for the effect of percent 15 to 29 on (ln) homicide rates – Long Series sample (since 1960). Shown are the coefficients from the quantile regression model with fixed effects for the Long Series Sample. Coefficients are exponentiated and correspond to the average proportional change in the homicide rate from a one-unit increase in the corresponding independent variable. In parenthesis are standard errors clustered by country. ***p < 0.001; **p < 0.01; *p < 0.05.

	Fixed Effects	*Quantile Fixed Effects Models*				
		$\tau = 0.1$	$\tau = 0.25$	$\tau = 0.5$	$\tau = 0.75$	$\tau = 0.9$
**Percent 15 to 29**	**1.054*****	**1.085*****	**1.071*****	**1.054*****	**1.050*****	**1.050*****
	**(0.014)**	**(0.005)**	**(0.007)**	**(0.008)**	**(0.008)**	**(0.010)**
Percent Male	1.125	1.025	1.079	1.095*	1.045	0.988
	(0.075)	(0.042)	(0.045)	(0.045)	(0.060)	(0.088)
Gini Index	0.967	0.954***	0.959***	0.960***	0.966**	0.988
	(0.019)	(0.005)	(0.006)	(0.007)	(0.011)	(0.013)
GDP per Cap (1k)	0.997	1.002	1.000	0.996	0.993*	0.995
	(0.006)	(0.002)	(0.002)	(0.002)	(0.003)	(0.005)
Percent Urban	1.022*	1.021***	1.023***	1.026***	1.028***	1.026***
	(0.009)	(0.003)	(0.003)	(0.003)	(0.002)	(0.004)
Observations	1,136	1,136	1,136	1,136	1,136	1,136
