# Supplementary material for: The contribution of age structure to the international homicide decline
Source: PLoS One. 2019 Oct 9;14(10):e0222996. doi: 10.1371/journal.pone.0222996 (PMC6784918; doi:10.1371/journal.pone.0222996)
Supplement: S9 Table — Shown are the results from fixed effects regression models estimating the natural log of homicide rates as a function of percent 15 to 24 and other control variables. Coefficients are exponentiated and correspond to the average proportional change in the homicide rate from a one-unit increase in the corresponding independent variable. In parenthesis are robust standard errors clustered by country. ***p < 0.001; **p < 0.01; *p < 0.05. (PDF) [file pone.0222996.s018.pdf]

S9 Table. Sensitive analysis - Fixed effects models for the average effect of percent 15 to 24 on homicide rate. Shown are the results from fixed effects regression models estimating the natural log of homicide rates as a function of percent 15 to 24 and other control variables. Coefficients are exponentiated and correspond to the average proportional change in the homicide rate from a one-unit increase in the corresponding independent variable. In parenthesis are robust standard errors clustered by country. ***p < 0.001; **p < 0.01; *p < 0.05.

	High Coverage Sample		Long Series Sample			
	Since 1990	Since 1990	Since 1960	Since 1960	Since 1990	Since 1990
**Percent 15 to 24**	**1.063**** **(0.017)**	**1.030 (0.018)**	**1.055**** **(0.018)**	**1.063***** **(0.017)**	**1.080***** **(0.023)**	**1.057*** **(0.026)**
Percent Male		1.036 (0.050)		1.113 (0.080)		1.153 (0.074)
Gini Index		0.987 (0.016)		0.961* (0.019)		0.959 (0.039)
GDP per Cap (1k)		0.970** (0.010)		0.996 (0.006)		0.984 (0.010)
Percent Urban		1.009 (0.009)		1.024* (0.009)		1.011 (0.017)
Observations	2,283	2,283	1,136	1,136	662	662
Countries	126	126	26	26	26	26
R^2	0.052	0.129	0.093	0.242	0.137	0.252
F Statistic	117.427***	63.913***	113.761***	70.405***	100.772***	42.528***
