# Supplementary material for: The contribution of age structure to the international homicide decline
Source: PLoS One. 2019 Oct 9;14(10):e0222996. doi: 10.1371/journal.pone.0222996 (PMC6784918; doi:10.1371/journal.pone.0222996)
Supplement: S10 Table — Shown are the results from fixed effects regression models estimating the natural log of homicide rates as a function of percent 15 to 29 and other control variables. Huber weights were used to reduce the influence of observations with high residuals on the regression estimates. Coefficients are exponentiated and correspond to the average proportional change in the standard deviation of the homicide rate from a one-unit increase in the standard deviation of the corresponding independent variable. In parenthesis are robust standard errors clustered by country. ***p < 0.001; **p < 0.01; *p < 0.05. (PDF) [file pone.0222996.s019.pdf]

S10 Table. Sensitive analysis – Robust regression models with fixed effects (Huber weights) for the average effect of percent 15 to 29 on homicide rates. Shown are the results from fixed effects regression models estimating the natural log of homicide rates as a function of percent 15 to 29 and other control variables. Huber weights were used to reduce the influence of observations with high residuals on the regression estimates. Coefficients are exponentiated and correspond to the average proportional change in the standard deviation of the homicide rate from a one-unit increase in the standard deviation of the corresponding independent variable. In parenthesis are robust standard errors clustered by country. ***p < 0.001; **p < 0.01; *p < 0.05.

	High Coverage Sample		Long Series Sample			
	Since 1990	Since 1990	Since 1960	Since 1960	Since 1990	Since 1990
**Percent 15 to 29**	1.053*** (0.013)	1.019 (0.014)	1.055*** (0.011)	1.055*** (0.013)	1.074*** (0.014)	1.056** (0.020)
Percent Male		1.053 (0.047)		1.08 (0.075)		1.073 (0.081)
Gini Index		0.989 (0.015)		0.973 (0.018)		0.976 (0.033)
GDP per Cap (1k)		0.970*** (0.009)		0.997 (0.005)		0.985 (0.009)
Percent Urban		1.009 (0.009)		1.021* (0.009)		1.014 (0.015)
Observations	2,283	2,283	1,136	1,136	662	662
Countries	126	126	26	26	26	26
R^2	0.052	0.124	0.132	0.257	0.192	0.275
F Statistic	439.712***	142.676***	306.204***	124.395***	255.925***	74.234***
