# Supplementary material for: The contribution of age structure to the international homicide decline
Source: PLoS One. 2019 Oct 9;14(10):e0222996. doi: 10.1371/journal.pone.0222996 (PMC6784918; doi:10.1371/journal.pone.0222996)
Supplement: S11 Table — Shown are the results from fixed effects regression models estimating the natural log of homicide rates as a function of percent 15 to 29 and other control variable using the Combined WHO and UNODC Series since 1960, compared to only WHO data since 1960 for the 26 countries of the Long Series Sample. Coefficients are exponentiated and correspond to the average proportional change in the homicide rate from a one-unit increase in the corresponding independent variable. In parenthesis are robust standard errors clustered by country. ***p < 0.001; **p < 0.01; *p < 0.05. (PDF) [file pone.0222996.s020.pdf]

S11 Table. Sensitive analysis comparing results of the Long Series sample using the combined UN & WHO series, against the WHO series alone. Shown are the results from fixed effects regression models estimating the natural log of homicide rates as a function of percent 15 to 29 and other control variable using the Combined WHO and UNODC Series since 1960, against only WHO data since 1960 for the 26 countries of the Long Series Sample. Coefficients are exponentiated and correspond to the average proportional change in the homicide rate from a one-unit increase in the corresponding independent variable. In parenthesis are robust standard errors clustered by country. ***p < 0.001; **p < 0.01; *p < 0.05.

	Combined Series		WHO Only	
	Since 1960	Since 1960	Since 1960	Since 1960
**Percent 15 to 29**	**1.053*****<br>**(0.011)**	**1.054*****<br>**(0.014)**	**1.062*****<br>**(0.012)**	**1.053*****<br>**(0.015)**
Percent Male		1.125<br>(0.075)		1.117<br>(0.077)
Gini Index		0.967<br>(0.019)		0.965<br>(0.019)
GDP per Cap (1k)		0.997<br>(0.006)		0.993<br>(0.005)
Percent Urban		1.022*<br>(0.009)		1.021*<br>(0.009)
Observations	1,136	1,136	1,127	1,127
R^2	0.132	0.259	0.166	0.276
F Statistic	168.597***	77.294***	218.477***	83.606***
