# Supplementary material for: The contribution of age structure to the international homicide decline
Source: PLoS One. 2019 Oct 9;14(10):e0222996. doi: 10.1371/journal.pone.0222996 (PMC6784918; doi:10.1371/journal.pone.0222996)
Supplement: S12 Table — Shown are the fixed effects regression estimates using the High Cover and Long Series Sample using all observed data on Homicide Rate and Percent 15 to 29. Coefficients are exponentiated and correspond to the average proportional change in the homicide rate from a one-unit increase in the corresponding independent variable. In parenthesis are standard errors clustered by country. ***p < 0.001; **p < 0.01; *p < 0.05. (PDF) [file pone.0222996.s021.pdf]

S12 Table. Sensitive analysis restricting the bivariate models to the fully controlled sample of observations. Shown are the fixed effects regression estimates using the High Cover and Long Series Sample using all observed data on Homicide Rate and Percent 15 to 29. Coefficients are exponentiated and correspond to the average proportional change in the homicide rate from a one-unit increase in the corresponding independent variable. In parenthesis are standard errors clustered by country. ***p < 0.001; **p < 0.01; *p < 0.05.

	High Coverage Sample			Long Series Sample		
	Since 1990	Since 1990	Since 1990	Since 1990	Since 1960	Since 1960
**Percent 15 to 29**	**1.050*****<br>**(0.014)**	**1.018**<br>**(0.015)**	**1.038****<br>**(0.013)**	**1.053*****<br>**(0.011)**	**1.054*****<br>**(0.014)**	**1.051*****<br>**(0.014)**
Percent Male		1.032<br>(0.053)			1.125<br>(0.075)	
Gini Index		0.989<br>(0.016)			0.967<br>(0.019)	
GDP per Cap (1k)		0.969**<br>(0.010)			0.997<br>(0.006)	
Percent Urban		1.008<br>(0.009)			1.022*<br>(0.009)	
Observations	2,283	2,283	2,558	1,136	1,136	1,621
Countries	126	126	135	26	26	26
R^2	0.052	0.125	0.032	0.132	0.259	0.08
F Statistic	117.915***	61.538***	80.688***	168.597***	77.294***	139.432***
