# Supplementary material for: The contribution of age structure to the international homicide decline
Source: PLoS One. 2019 Oct 9;14(10):e0222996. doi: 10.1371/journal.pone.0222996 (PMC6784918; doi:10.1371/journal.pone.0222996)
Supplement: S13 Table — Shown are the results from fixed effects regression models estimating the natural log of homicide rates as a function of the absolute size of the population 15 to 29 (in millions of individuals) and other control variables. Coefficients are exponentiated and correspond to the average proportional change in the homicide rate from a one-unit increase in the corresponding independent variable. In parenthesis are robust standard errors clustered by country. ***p < 0.001; **p < 0.01; *p < 0.05. (PDF) [file pone.0222996.s022.pdf]

S13 Table. Sensitive analysis - Fixed effects models for the average effect of the absolute size of the population 15 to 29 on homicide rate. Shown are the results from fixed effects regression models estimating the natural log of homicide rates as a function of the absolute size of the population 15 to 29 (in millions of individuals) and other control variables. Coefficients are exponentiated and correspond to the average proportional change in the homicide rate from a one-unit increase in the corresponding independent variable. In parenthesis are robust standard errors clustered by country. ***p < 0.001; **p < 0.01; *p < 0.05.

	High Coverage Sample		Long Series Sample			
	Since 1990	Since 1990	Since 1960	Since 1960	Since 1990	Since 1990
**Absolute Pop 15 to 29 (1 Million)**	0.999<br>(0.003)	0.999<br>(0.003)	1.014<br>(0.016)	1.004<br>(0.020)	1.031<br>(0.040)	1.033<br>(0.034)
Percent Male		1.049<br>(0.050)		1.168<br>(0.084)		1.189^{*}<br>(0.074)
Gini Index		0.988<br>(0.017)		0.957^{*}<br>(0.017)		0.977<br>(0.040)
GDP per Cap (1k)		0.966^{***}<br>(0.010)		0.988^{*}<br>(0.006)		0.976^{*}<br>(0.011)
Percent Urban		1.007<br>(0.009)		1.021^{*}<br>(0.010)		1.006<br>(0.017)
Observations	2,283	2,283	1,136	1,136	662	662
Countries	126	126	26	26	26	26
R^2	0.0002	0.120	0.010	0.183	0.013	0.226
F Statistic	0.440	58.819^{***}	11.273^{***}	49.524^{***}	8.601^{**}	36.916^{***}
