# Supplementary material for: The contribution of age structure to the international homicide decline
Source: PLoS One. 2019 Oct 9;14(10):e0222996. doi: 10.1371/journal.pone.0222996 (PMC6784918; doi:10.1371/journal.pone.0222996)
Supplement: S15 Table — Shown are the results from fixed effects regression models estimating the natural log of homicide rates as a function of the size of the youth cohort relative to older age groups, and as a function of males 15 to 29 years over the total population. The model including the original measure of percent youth (ages 15 to 29 over the total population) is in the and fifth columns. To account for differences in the effective range of each age measure and to enable a direct comparison between coefficients, all measures included in the model were standardized. Coefficients are exponentiated and correspond to the average proportional change in the standard deviation of the homicide rate from a one-unit increase in the standard deviation of the corresponding independent variable. In parenthesis are robust standard errors clustered by country. ***p < 0.001; **p < 0.01; *p < 0.05. (PDF) [file pone.0222996.s024.pdf]

S15 Table. Sensitive analysis - Fixed effects models for the average effect of youth cohort size relative to older age groups. Shown are the results from fixed effects regression models estimating the natural log of homicide rates as a function of the size of the youth cohort relative to older age groups, and as a function of males 15 to 29 years over the total population. The model including the original measure of percent youth (ages 15 to 29 over the total population) is in the and fifth columns. To account for differences in the effective range of each age measure and to enable a direct comparison between coefficients, all measures included in the model were standardized. Coefficients are exponentiated and correspond to the average proportional change in the standard deviation of the homicide rate from a one-unit increase in the standard deviation of the corresponding independent variable. In parenthesis are robust standard errors clustered by country. ***p < 0.001; **p < 0.01; *p < 0.05.

	High Coverage Sample				Long Series Sample			
	Since 1990	Since 1990	Since 1990	Since 1990	Since 1960	Since 1960	Since 1960	Since 1960
**Ages 15-29 over Total**	**1.044**<br>**(0.037)**				**1.134*****<br>**(0.034)**			
**Ages 15-29 over 30-59**		**1.075**<br>**(0.066)**				**1.138**<br>**(0.100)**		
**Ages 15-24 over 25-59**			**1.105**<br>**(0.057)**				**1.121**<br>**(0.093)**	
**Males 15-29 over Total**				**1.048**<br>**(0.036)**				**1.178*****<br>**(0.035)**
Percent Male	1.065<br>(0.104)	1.074<br>(0.098)	1.075<br>(0.094)		1.262<br>(0.149)	1.236<br>(0.162)	1.245<br>(0.159)	
Gini Index	0.925<br>(0.113)	0.913<br>(0.113)	0.901<br>(0.113)	0.926<br>(0.114)	0.794<br>(0.129)	0.753*<br>(0.136)	0.737*<br>(0.133)	0.792<br>(0.126)
GDP per Cap (1k)	0.698**<br>(0.116)	0.689***<br>(0.112)	0.695***<br>(0.109)	0.695**<br>(0.113)	0.961<br>(0.065)	0.904<br>(0.083)	0.903<br>(0.083)	0.97<br>(0.064)
Percent Urban	1.17<br>(0.180)	1.228<br>(0.190)	1.261<br>(0.194)	1.155<br>(0.180)	1.539*<br>(0.181)	1.703***<br>(0.157)	1.693***<br>(0.159)	1.463*<br>(0.189)
Observations	2,283	2,283	2,283	2,283	1,136	1,136	1,136	1,136
Countries	126	126	126	126	26	26	26	26
R^2	0.125	0.124	0.128	0.122	0.259	0.206	0.201	0.247
F Statistic	61.538***	60.716***	63.085***	74.827***	77.294***	57.338***	55.596***	90.562***
